# Research Progress of Thermally Conductive Rubber Composites for Tire Heat Dissipation

**DOI:** 10.3390/polym17233197

**Published:** 2025-11-30

**Authors:** Suling Chang, Zhihao Wang, Xiaoyao Wang, Tingxi Dong, Si Li, Haishan Yin

**Affiliations:** 1 College of Electromechanical and Engineering, Qingdao University of Science and Technology, Qingdao 266100, China; changsuling@163.com (S.C.); 18253782539@163.com (Z.W.); 19710531057@163.com (X.W.); 18591734852@163.com (T.D.); lisi199806@163.com (S.L.); 2 Shanxi Yanchang Petroleum Group Rubber Co., Ltd., Xianyang 712000, China

**Keywords:** thermal conductivity, rubber composites, fillers, tires

## Abstract

In light of the increasing demand for improved thermal performance within the tire industry, research on thermally conductive rubber composites has become a significant focus of interest. This paper provides a comprehensive overview of the most recent research findings on thermally conductive rubber composites, specifically for tire heat-dissipation applications. First, the thermal-conductivity mechanism of rubber-based composites is elaborated in detail, and the influencing factors of heat dissipation and thermal conductivity in tire rubber are systematically analyzed. The role of various thermally conductive fillers in tire heat dissipation and their applications is highlighted, and the thermal conductivities of these fillers and their effects in practical tire applications are compared. In addition, the distribution of fillers is optimized by combining experimental studies with simulation methods (e.g., molecular dynamics simulation) to provide a scientific basis for tire design. Finally, this paper summarizes the main challenges currently faced by rubber composites in tire applications, including material costs, filler and matrix dispersion, and thermal resistance. It also proposes the potential future development direction of thermally conductive rubber composites in tire applications.

## 1. Introduction

The rapid advancement of microelectronics technology is increasing the demand for thermally conductive materials. Compared with other traditional materials, polymers and their composites have become a popular choice in engineering due to their low cost, easy processing, corrosion resistance, and versatility [1,2]. However, their thermal conductivity is usually in the range of 0.1 to 0.5 W/(m·K) [3,4], a characteristic that may lead to poor heat dissipation and localized overheating. Therefore, enhancing the thermal conductivity of polymer composites has become a new trend in the current composites industry [5].

Tire performance requirements are diverse. This requires tires to have good mechanical properties and wear resistance and to provide efficient heat dissipation in complex operating environments. However, conventional rubber materials are poor heat conductors, and heat build-up under dynamic conditions may lead to an uneven temperature distribution within the tire, affecting its safety and service life. Tires undergo substantial low-frequency mechanical deformation during extended use, driven by internal friction at the molecular level among fillers, between fillers and rubber, and within the rubber components themselves. This process generates significant heat, which is difficult to dissipate effectively due to the inherent poor thermal conductivity of rubber elastomers [6,7].

**Figure 1:**
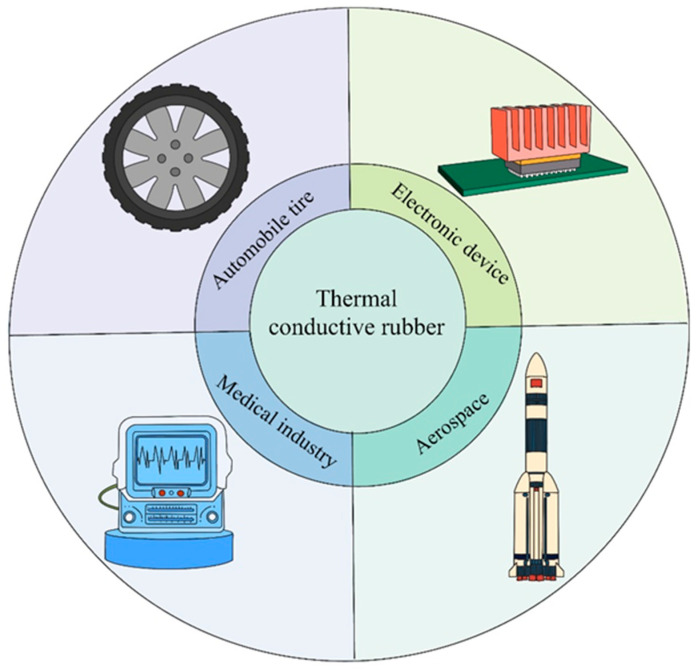
Thermally conductive rubber and its composite material application introduction.

Thermally conductive rubber, with its high thermal conductivity, is commonly used to replace ordinary rubber in applications involving heat transfer and dissipation. Meanwhile, due to its sound insulation and high-temperature resistance, it is widely used in aerospace, electronic equipment, biomedical, and automotive industries and other fields (Figure 1). At present, the research of polymer materials such as rubber is mainly focused on improving their thermal conductivity by incorporating high thermal conductivity fillers into the matrix [8]. As an essential branch of polymers, one of the key research topics in rubber research is to improve the heat resistance and thermal conductivity of rubber materials.

Despite the remarkable progress in this area of research, the literature on thermally conductive rubber remains relatively scarce. Although rubber composites have been widely used in tires, there are also relatively few systematic summaries of their thermal conductivity. In view of the increasing importance of heat dissipation in the tire industry, reducing tire driving temperature and improving tire heat dissipation are key to prolonging tire life and ensuring driving safety. Based on this, this paper summarizes recent advances in thermally conductive rubber composites for tire heat-dissipation applications. First, the thermal-conductivity mechanisms of rubber and its composites are explained; on this basis, the thermophysical properties of tires are analyzed, and the various factors affecting the thermal conductivity of rubber are reviewed, with emphasis on the application of different thermally conductive fillers in tire thermal management. Subsequently, the current challenges faced by rubber composites in tire applications are discussed, and the possibility of optimizing filler design through simulation, such as molecular dynamics, is explored. Finally, the prospects for rubber composites in tires are explored, aiming to provide constructive ideas for their broader application.

## 2. Thermal Conductivity Mechanism

The basic modes of heat transfer include conduction, convection, and radiation [9]. Heat conduction presupposes the transfer of heat, realized by the absence of macroscopic motion within the medium and of relative displacement, which depends on the thermal motion of microscopic particles within the object. Heat conduction follows Fourier’s law (1), which applies to isotropic materials.

*q* = −*λ* grad(t) (1)

In this equation, *q* represents the heat flow density (W/m^2^), defined as the amount of heat transferred through a unit area in a unit time; *λ* is the thermal conductivity coefficient (W/(m·K), which indicates the ability of a substance to transfer heat under a temperature gradient, and is a physical quantity that characterizes the strength of an object’s thermal conductivity. Grad t represents the temperature gradient at a given point in space (K/m), and the negative sign indicates that the direction of heat transfer is opposite to the direction of increasing temperature [10].

There are differences in the form and process of heat transfer in different states and conditions. It has been shown that heat conduction occurs primarily in solids. The microscopic particle medium for heat conduction mainly includes molecules, phonons, electrons, and photons. For solids, thermal conductivity is primarily realized by both lattice vibration and diffusion of free electrons [11]. In crystalline materials, thermal conductivity is primarily carried out through the thermal vibration of the grains, and the thermal conductivity medium is mostly phonons. In non-crystalline materials such as rubber, on the other hand, due to their disordered structure, the arrangement of molecules or atoms shows an irregular, haphazard distribution, and thermal vibration occurs around a fixed position. Although the heat carriers of amorphous materials are also phonons, due to the irregularity of their structure, there are a large number of defects on the surface (e.g., grain boundaries), which leads to scattering of phonons during propagation and affects the regular transport, thus making the thermal conductivity *λ* low [12]. In metals, thermal conductivity results from collisions and interactions between free electrons. The thermal conductivity of a material is a complex phenomenon that results from the combined thermal conductivities of all its internal microscopic particles.

The contribution of heat transfer carriers varies significantly from substance to substance, with one heat transfer carrier usually dominating [13]. In polymers, phonons are considered the primary heat transfer carriers [14]. Phonons are regarded as quantized lattice vibrational modes that follow the Borel-Einstein particle distribution and exhibit wave-particle duality. The thermal conductivity of rubber is generally low, mainly because most rubber materials are usually saturated systems, and there are almost no free electrons. In addition, factors such as the relatively weak forces between rubber molecules and the disordered molecular chain structure contribute to the low thermal conductivity.

Currently, three main theories can effectively explain the thermal conductivity behavior of filled thermally conductive rubber and its influencing factors: the thermal conductivity path theory, the thermal penetration theory, and the thermoelastic coefficient theory [15,16,17].

**Figure 2:**
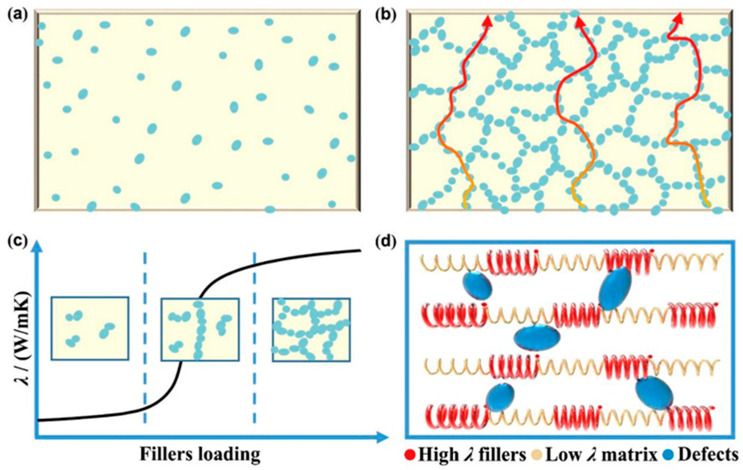
(**a**) “Sea-island” in low fillers loading; (**b**) Thermal conduction paths in high fillers loading; (**c**) Percolation phenomenon; (**d**) Thermoelastic coefficient theory. Reprinted from [26], Copyright (2020), with permission from Elsevier.

The theory of thermally conductive pathways has been widely recognized. The theory states that the overlapping and synergistic action of thermally conductive fillers in the polymer matrix forms a continuous thermally conductive path [18,19]. The number of fillers significantly affects thermal conductivity. In the rubber matrix, at low filler concentrations, particles are sparsely dispersed, making it difficult to form a continuous thermal conductive network. Heat still needs to be transferred through the insulating rubber matrix, which is equivalent to “heat flux having to detour through a low thermal conductivity medium,” resulting in limited improvement in overall thermal conductivity, resulting in a “sea-island” two-phase system (Figure 2a). As a result, the thermal conductivity of the composite is low. With the gradual increase in the content of thermally conductive fillers, the contact between them becomes tighter, leading to the formation of a complete thermally conductive path or network. At this point, the heat flow passes along the heat-conducting channels, thus reducing the overall thermal resistance (Figure 2b) [20]. Theoretically, when a thermally conductive network is formed, the thermal conductivity of polymer composites will significantly increase and exhibit osmosis. However, the thermal conductivity of most thermal conductive fillers and polymers only differs by 10 to 10^3^ times. The thermal conductivity of polymers seldom changes abruptly over a wide range of filler dosages. No obvious transition point for over-permeation has been found, so the theory of thermal permeation remains controversial [21]. However, for fillers with very high intrinsic thermal conductivity, permeation does occur (Figure 2c) [22,23,24].

Unlike the two theories mentioned above, the thermoelastic coefficient theory combines the complex principles of classical vibrational mechanics and elastic mechanics. Some researchers believe that *λ* is analogous to the thermoelastic coefficient in phonon propagation. In other words, the higher the *λ*-value of the composite, the higher the thermoelastic coefficient and phonon propagation efficiency (Figure 2d) [25]. Therefore, the enhancement of the λ value in rubber is regarded as a consequence of the synergistic interaction between high thermal conductivity fillers and a low thermal conductivity matrix. According to the model, during propagation, phonons will be scattered by vibrations and fluctuations at the interface between two phases with different thermoelastic coefficients, thereby hindering heat conduction.

In summary, these three theories collectively emphasize that the selection, quantity, and uniform distribution of fillers, as well as the reduction in interfacial thermal resistance, are prerequisites for the formation of effective thermal conductivity pathways. To increase the thermal conductivity of polymers, a continuous, comprehensive thermally conductive network must be built. Thus, optimizing the development of thermally conductive networks within the system will be the primary goal of future studies on the theory of thermally conductive rubbers.

## 3. Tire Rubber Heat Dissipation and Thermal Conductivity Influencing Factors

Heat is one of the most essential damaging factors for rubber products. Heat accumulation not only significantly reduces the mechanical properties of rubber products but also accelerates material aging and shortens fatigue life [27]. The factors affecting heat generation in rubber are pretty complex, including the Payne effect, the Mullins effect, interfacial interactions, bound rubber, cross-linking density, and fillers [28]. Tires are everyday rubber composite products. It is estimated that the proportion of rubber in tires is about 40–50% [29]. The heat generation mechanism in tires can be divided into two main parts. In addition to the hysteresis heat generation caused by the strain lagging behind the stress mentioned above, frictional work due to direct contact between the tire and the road surface is also a cause of the temperature rise. Compared with heat loss, friction heat accounts for a smaller proportion.

Thermophysical properties (thermal conductivity, thermal diffusion coefficient, and specific heat) are key parameters for evaluating a material’s heat dissipation [6]. The high temperatures generated during vulcanization may cause tire damage. Therefore, improving the thermal diffusivity of rubber can effectively reduce heat accumulation [30]. In addition, improving tire thermal conductivity is an effective way to enhance heat dissipation. In addition to these direct indicators, tan δ indirectly reflects the thermal behavior of materials under dynamic loading by characterizing their energy dissipation properties. rubbers with high tan δ values lead to the conversion of mechanical energy into thermal energy under dynamic loading, which exacerbates the accumulation of heat in the rubber [31]. To mitigate tire overheating, in addition to focusing on theory and modeling, an in-depth study of the rubber formulation is required. The research by Abey Gunawardane et al. [32] indicates that effectively controlling heat accumulation is crucial for reducing the risk of tire blowouts and improving the problem of shortened fatigue life caused by material performance degradation. From the perspective of manufacturing processes, adding high thermal conductivity fillers (such as flake graphite powder) to materials can help shorten the vulcanization time, thereby enhancing production efficiency and energy utilization efficiency. Wu et al. [33] pointed out that the heat generated by hysteresis loss during the rolling process of tires can cause a significant increase in internal temperature, with the highest internal temperature reaching 35.28 °C, which is higher than the surface temperature. If the accumulation of heat is not effectively controlled, it may lead to accelerated tread wear or even a blowout risk, seriously affecting landing safety. In terms of durability, the strain amplitude has a significant impact on temperature rise. When the strain increases from 0.20 to 0.36, the stable temperature rises by 53%, indicating that high temperatures accelerate the aging and fatigue of rubber materials and shorten their service life. In terms of energy efficiency, the increase in rolling resistance caused by heat accumulation will further increase energy loss.

**Figure 3:**
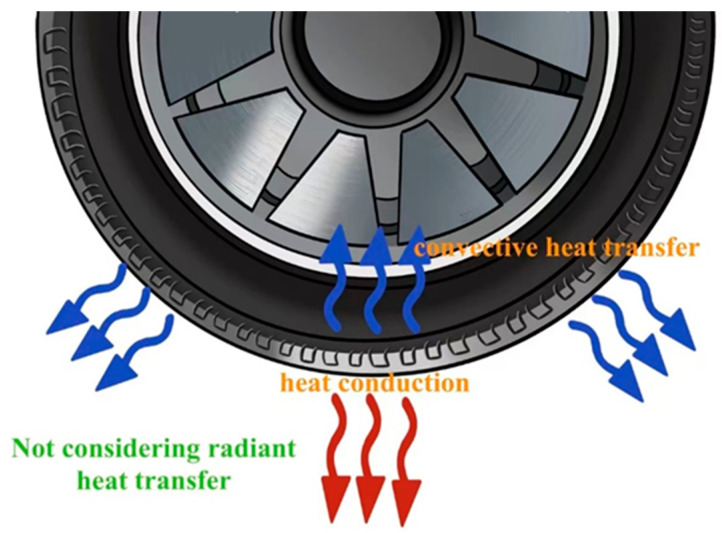
Mechanism of heat transfer between tires and their surroundings.

There are three primary forms of tire heat dissipation: convective heat transfer between the tire surface and the surrounding air, heat conduction between the tread and the ground, and radiative heat transfer between the tire and its surroundings. Typically, heat radiation from the tire to its surroundings is not considered [34]. The primary heat transfer mechanism of a tire is shown in Figure 3. Due to the influence of many factors, the heat transfer in the convection process is more complicated. Scholars at home and abroad have conducted in-depth research on convective heat transfer at the tire surface through theoretical analysis, numerical simulation, and experimental validation, achieving fruitful results that have significantly enhanced tire heat dissipation performance. Similarly, the use of thermally conductive rubber composites can dissipate heat more effectively, preventing tread temperatures from becoming too high.

**Figure 4:**
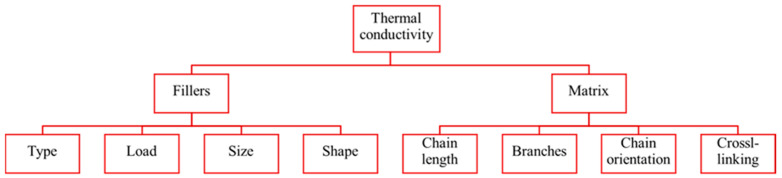
The main factors influencing the thermal conductivity of rubber composites.

A combination of factors determines the thermal conductivity of rubber composites. Figure 4 briefly summarizes the effect of filler and polymer matrix on their thermal conductivity. In addition, temperature and humidity also affect the λ value [26,35,36,37].

### 3.1. Types of Fillers

The effect of different types of fillers in enhancing the thermal conductivity of composites varies significantly. Thermally conductive fillers can be mainly categorized into the following groups: metal particle fillers (e.g., Ag, Cu, Al, and Ni [38]), carbon-based materials (including graphite, carbon fibers, nano-diamond [39], carbon nanotubes, carbon black, and graphene), and ceramic materials (e.g., BN, AlN, Al_2_O_3_, ZnO and SiC [40]). Table 1 presents λ values for different types of thermal-conductive fillers [26,41,42,43,44,45,46,47,48].

### 3.2. Fillers Shape

There are significant differences in the contact area and the arrangement of thermal-conductive fillers with different shapes. The thermal conductivity of composites is significantly influenced by the shape of the fillers [49,50]. On the one hand, the shape of the filler affects interfacial properties with the rubber matrix, and a good filler morphology can improve contact between the filler and the matrix, thereby effectively reducing the contact thermal resistance within the material. On the other hand, the shape of the filler can also affect the heat conduction path in the composite material. These theoretical principles are well demonstrated in the comparative study by Li et al. [51], who investigated the effects of spherical Al_2_O_3_, irregularly shaped AlN, and two-dimensional sheet BN on the properties of silicone rubber. Their research found that the two-dimensional BN fillers were the most effective in constructing continuous thermal conduction networks, thereby significantly enhancing the thermal conductivity of the composites.

The filler’s dimensions strongly influence the thermal conductivity. A larger filler reduces the direct contact area between the filler and the matrix at the same volume, thereby lowering the interfacial thermal resistance and enhancing thermal conductivity [52,53]. However, in some cases, the introduction of minor fillers can also improve the composite’s thermal conductivity [54,55]. Therefore, at present, the industry usually uses different particle-size fillers in combination.

The aspect ratio is a significant factor influencing the thermal conductivity of rubber composites [56]. High aspect ratio fillers, including linear, sheet, and fibrous fillers, demonstrate superior thermal conductivity compared to spherical fillers. Research has found that compared with low aspect ratio fillers, graphene with a high aspect ratio can more significantly enhance the thermal conductivity of composite materials. Its enhancement mechanism is attributed to the fact that a high aspect ratio strongly restricts the random aggregation and orientation of the filler, thereby inducing its self-assembly to form a highly ordered thermal conduction network [57]. However, excessively high aspect ratios can sometimes adversely affect thermal conductivity. For instance, Evign et al. [58] found that when the aspect ratio of carbon nanotubes (CNTS) exceeds 300:1, the thermal conductivity of the composite material actually decreases by 12% to 15%. The reason for this is that an excessively high aspect ratio can cause severe agglomeration of the filler, which not only forms a thermal conduction path but also introduces a large amount of interinterface thermal resistance, thereby weakening the overall thermal conductivity. Based on this, the research suggests that in polymer-based composites, the aspect ratio of carbon nanotubes being controlled within the range of 20 to 100 is most conducive to the improvement of thermal conductivity.

### 3.3. Fillers Loading

Typically, the thermal conductivity of rubber composites increases with increasing thermally conductive filler loading [59]. To create an efficient thermal conduction pathway, fillers must be incorporated in precise quantities. However, excessive filler content can increase weight and cost while reducing the composite’s mechanical properties. Studies have shown that under high filler content, due to weak polymer-filler interactions and strong filler-filler interactions, the performance of BR composites is worse than that of SSBR. It has been confirmed that the quasi-static mechanical properties first increase and then decrease with increasing filler content, indicating the existence of an optimal filler amount. It is particularly noted that high filler content leads to a sharp increase in the elastic modulus of the elastomer, severely affecting processing performance and practical applications [60].

### 3.4. Filler Functionalization

Interfacial compatibility between the filler and the rubber matrix is a key factor in achieving excellent overall performance in composites. The functional treatment can effectively reduce the interface thermal resistance, improve the filler dispersion and binding force, and optimize the phonon transport channel. Researchers have previously used a variety of functionalization treatments on thermally conductive fillers, including acid and alkali treatments [61], solvent-assisted ball milling [62], coupling-agent modification [63], and surface coating methods [64].

### 3.5. Fillers Orientation

To improve the thermal conductivity of composites, many researchers have focused on optimizing the controlled dispersion of thermally conductive fillers [65]. By precisely controlling the process parameters during processing, fillers can be made to adopt a specific orientation, thereby achieving anisotropic thermal conductivity. Currently, several methods have been attempted to adjust the orientation of fillers in the rubber matrix, including electric-field induction [66], magnetic-field induction [67], 3D printing [68], the template method [69], and electrostatic spinning [70].

In summary, the thermal conductivity of tire rubber is closely related to the filler. The use of thermally conductive fillers in tires helps improve heat dissipation efficiency, lower operating temperatures, and reduce thermal stress and aging. By optimizing the type, size, loading, functionalization, and orientation of the filler, heat dissipation in tires can be effectively supported, extending their service life and improving safety. In addition, special attention will be paid to aspect ratios when selecting fillers in the future. Fillers with high aspect ratios, such as graphene, significantly influence the formation of thermally conductive paths. Through rational design and selection of fillers, a balance between properties can be achieved, leading to the development of tire rubber matrix composites with a combination of excellent properties.

## 4. From Microelectronics to Tires: Applications and Challenges of Thermally Conductive Rubber Composites

From the development of highly thermally conductive interface materials (TIMs) to high-performance formulations for the tire industry, the demand for composite materials with excellent mechanical properties and high thermal conductivity continues to rise.

For tire rubber materials, achieving high thermal conductivity and low heat buildup while maintaining outstanding mechanical properties is an urgent challenge for the industry. It is not easy to integrate all the excellent properties of rubber materials through blending and compounding methods [71]. Based on high-thermal-conductivity fillers, this chapter reviews recent state-of-the-art approaches to improving the thermal conductivity of rubber composites using metallic, carbon-based, ceramic, and hybrid fillers, with a special focus on heat dissipation in tire rubber products. Solutions to address high tire temperatures are explored by analyzing aspects of the vulcanization process, thermal diffusivity, heat accumulation, and thermal conductivity. In addition, the chapter briefly introduces the application of bio-based fillers in rubber composites as a potential option for future “green tires”.

### 4.1. Metallic Materials

Metallic materials are used as common thermally conductive fillers with high wear resistance and strong stability. However, weak adhesion to the rubber matrix may reduce the composite’s overall thermal conductivity. Therefore, it is necessary to improve the interfacial bonding properties of metal fillers through surface modification. Commonly used thermally conductive metal particles include silver, copper, aluminum, and nickel. In addition to solid metals, liquid metals also have a wide range of applications in thermal conductivity and heat dissipation [72]. However, when metals are used as thermal conductive fillers, their excellent electrical conductivity is not applicable in some scenarios [73]. In products such as tires and insulating seals that require electrical isolation, the conductivity of metal fillers may cause leakage. At this point, modification methods such as surface coating need to be adopted to reduce their electrical conductivity while ensuring thermal conductivity, and to balance the compatibility of thermal conductivity and electrical conductivity.

Silver, copper, and aluminum have excellent thermal conductivity properties [72,73,74,75,76]. However, in industrial applications, it is often essential to modify the metal surface to enhance its compatibility with the rubber matrix, thereby optimizing phonon propagation and achieving high thermal conductivity in rubber composites. Rubber matrix is a commonly used composite matrix in industry, especially in the tire industry. Doping of natural rubber with aluminum powder can significantly increase its thermal conductivity. It was shown that the interfacial compatibility between aluminum powder and natural rubber (Al/NR) composites, after surface wet modification with the coupling agent Si69, was significantly improved, and their thermal conductivity increased from 0.25 W/m·K without modification to 0.47 W/m·K [77].

In a polydimethylsiloxane (PDMS) matrix, thermal conductivity can be enhanced by optimizing the size combination of alumina fillers. It was found that when 10 wt% of alumina powder was replaced with copper powder, the thermal conductivity reached a maximum of 2.466 W/m·K [78]. In addition, Tutika et al. [79] successfully prepared high-thermal-conductivity composites by filling metal solid particles (Fe, Ag, Au, Ni) and a liquid metal (gallium-indium alloy) into a silicone rubber matrix. It was shown that when the volume fraction of metal fillers (φ < 40%) was added to silicone rubber and the volume fraction of liquid metal (φ = 80%) was used, the thermal conductivity of the composites was significantly improved to 6.7 W/m·K.

The mechanical properties, such as modulus and fracture stress, are usually improved by adding metal fillers to rubber [80]. However, they also face challenges such as high density, susceptibility to corrosion, oxidation, and high cost, which somewhat limit their effectiveness and application in rubber composites. In addition, due to the conductive nature of metals, their incorporation into the rubber matrix may reduce the dielectric strength and electrical insulation of the composites, thereby limiting their application in electronic packaging. Therefore, the application scope of metal fillers as thermal conductive fillers alone is relatively limited. To enhance the overall performance, it is usually necessary to combine it with process improvements, such as using it in combination with other types of fillers. It is worth noting that the interfacial compatibility issue between metal fillers and rubber matrices may also have a negative impact on the mechanical properties of the material. For instance, in their study of iron and aluminum-filled styrene-butadiene rubber, Alam et al. [81] discovered that the presence of iron filler interacts with the sulfur vulcanization system. This interaction significantly diminishes the crosslinking density of the rubber matrix, leading to a reduction in key mechanical properties such as tensile strength, which can even fall below that of unfilled pure rubber. The decline in mechanical performance resulting from chemical incompatibility at the filler-matrix interface severely limits the applicability of this type of composite material in structural components that demand high levels of mechanical reliability and durability, such as tires and dynamic seals.

### 4.2. Carbon-Based Materials

Carbon-based fillers play a vital role in today’s technological and industrial fields. Carbon-based fillers have attracted significant attention for their high thermal conductivity, lightweight properties, and corrosion resistance [82,83]. Graphite, carbon nanotubes, graphene, and carbon fibers are the primary carbon-based fillers that significantly enhance the overall thermal conductivity of rubber matrices. However, the application of carbon-based fillers in tire thermal conductivity materials still faces some challenges and limitations.

**Figure 5:**
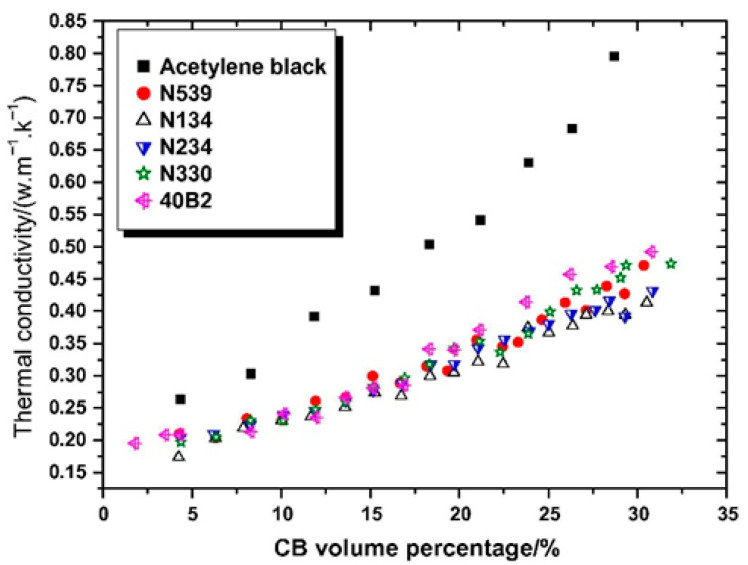
Influence of CB varieties on thermal conductivity of the composites. Reprinted from [85], Copyright (2019), with permission from Elsevier.

Carbon black (CB) has become an indispensable reinforcing agent in the rubber industry due to its cost-effectiveness, which significantly improves the hardness, strength, abrasion resistance, and thermal conductivity of rubber. It was found that adding carbon black to natural rubber significantly increased the Payne effect [84]. Song et al. [85] investigated the thermal conductivity of six different types of carbon black/rubber composites. They found that thermal conductivity increased with increasing filler content (Figure 5). In recent years, as environmental protection requirements have increased, the environmental pollution caused by carbon black in the tire production process cannot be ignored [86]. Therefore, carbon black is no longer able to meet the multifaceted requirements of rubber products.

**Figure 6:**
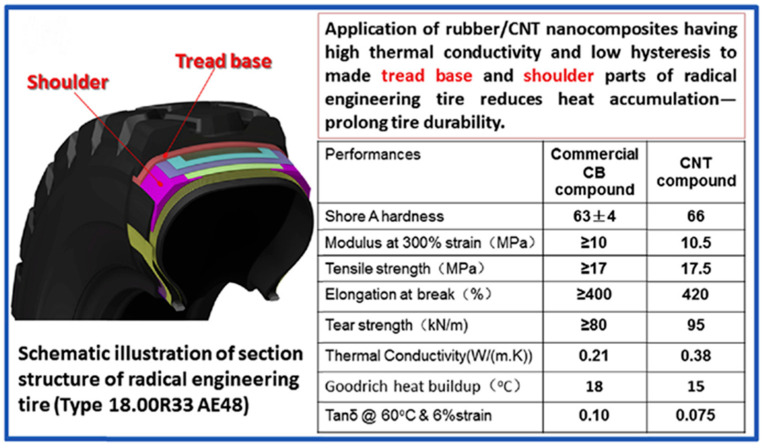
Thermal conductivity and heat buildup of rubber nanocomposites filled with carbon nanotubes. Adapted with permission from Ref. [87]: Copyright (2016) Elsevier.

Carbon nanotubes are often used as thermally conductive fillers in rubber composites due to their unique structure. Aiming at the problems of high entanglement in the preparation process of carbon nanotubes and difficulty in dispersing uniformly in the rubber matrix, Lu et al. [87] present a new method to achieve good dispersion of multi-walled carbon nanotubes (MWCNTs) in a rubber matrix. The obtained nanocomposites exhibit excellent mechanical properties, thermal conductivity, and good resistance to crack extension and fatigue (Figure 6). This approach successfully solves the problems of the tire industry in pursuing high performance, energy saving, and achieving sustainable mass production of engineered tires, while creating new opportunities for the widespread use of carbon nanotubes.

**Figure 7:**
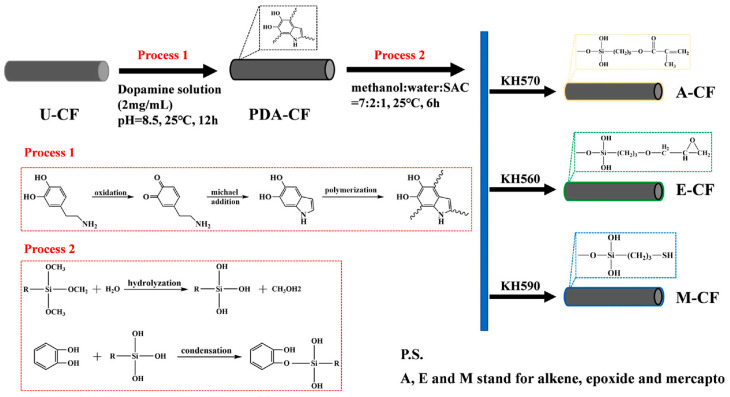
Process of silane functional modification of carbon fiber. Reprinted from [89], Copyright (2023), with permission from Elsevier.

Carbon fiber is mainly used as a reinforcing filler in polymers to optimize their mechanical properties, such as strength, stiffness and toughness. Meanwhile, its excellent thermal conductivity can also be attributed to the polymer matrix [88]. However, the lack of polar groups, such as hydroxyl and carboxyl groups, on the surface of carbon fibers reduces adhesion between the fibers and the rubber matrix. Therefore, it is particularly necessary to treat the surface of carbon fibers. Chen et al. [89] proposed a dual-modification method to incorporate carbon fibers into natural rubber. The specific modification process is shown in Figure 7. The results show that the enhancement of natural rubber properties using three coupling agents (KH560, KH570, and KH590) varies, with KH590 showing the most significant effect, increasing thermal conductivity by 71.3%.

**Figure 8:**
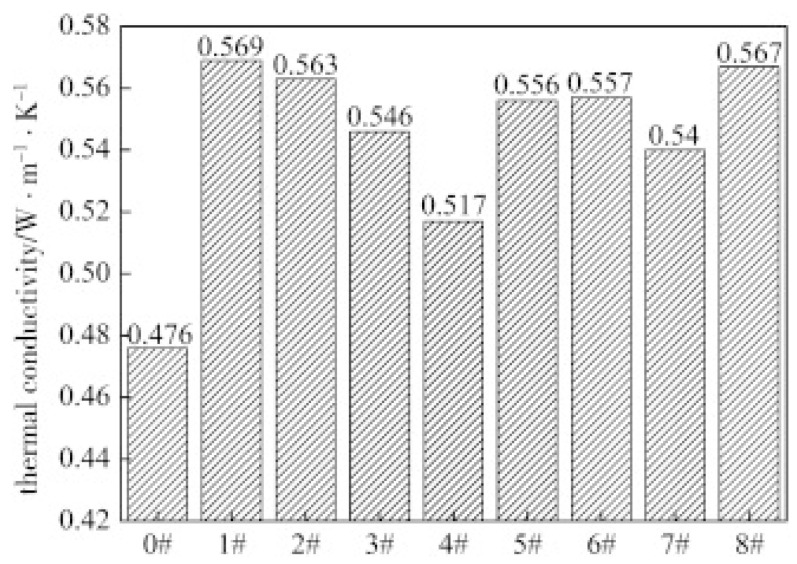
Thermal conductivity of graphite filled NR composites. Reprinted from [90], Copyright (2015), with permission from Elsevier.

Graphite is a conventional carbon-based filler that is inexpensive and, at the same time, endows rubber polymers with excellent heat transfer capability. Song et al. [90] developed modified graphite/natural rubber composites, using modified graphite as the filler via emulsion polymerization and polyacrylate modification. The findings indicated that when the ratio of methyl methacrylate to n-butyl acrylate was 1:1, the thermal conductivity reached a peak of 0.569 W/m·K, as illustrated in Figure 8. The incorporation of modified graphite increased the cross-linking density of the composites, extended scorch time, reduced vulcanization time, and enhanced mechanical properties. Consequently, these results suggest that this rubber composite holds promise for tire applications, offering a novel solution to improve tire heat dissipation.

Graphene, a two-dimensional material consisting of a single layer of carbon atoms, has attracted considerable attention in recent years due to its exceptional thermal conductivity and intrinsic charge mobility. Specifically, graphene/rubber composites have demonstrated the potential to enhance thermal conductivity even with minimal filler content [91]. However, dispersion in the rubber matrix is quite essential, and the vulcanization process during composite preparation also affects it. In addition, the high costs limit mass production. On the contrary, graphene derivatives, such as graphene oxide (GO) and reduced graphene oxide (rGO), can partially address the problem of homogeneous dispersion [92].

**Figure 9:**
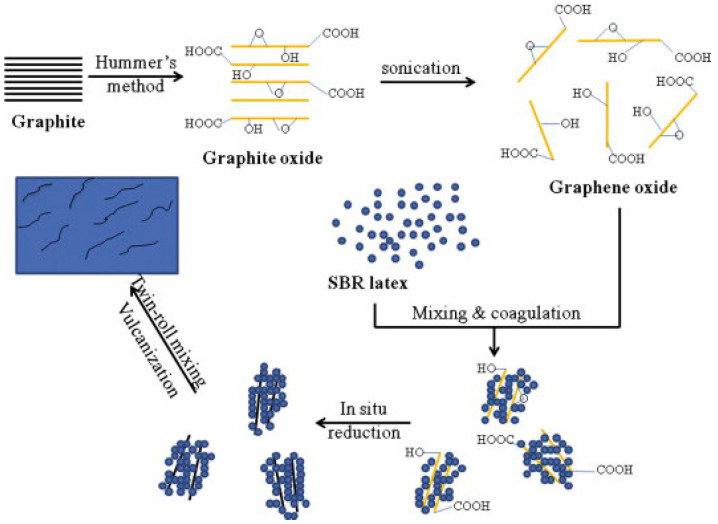
Preparation of GE/SBR nanocomposites. Reprinted from [93], Copyright (2014), with permission from Elsevier.

Xing et al. [93] investigated the effect of graphene (GE) nanofillers on the multifunctionality of styrene butadiene rubber (SBR). Given graphene’s tendency to agglomerate and poor dispersion in the rubber matrix, it needs to be modified to improve its compatibility with the rubber matrix. Therefore, the researchers prepared graphene/styrene-butadiene rubber nanocomposites using a modified latex blending method (Figure 9). Among them, the tensile strength increased nearly 11 times at the graphene addition of 7 phr. In addition, the composites also achieved significant progress in other properties. The modified graphene/SBR rubber nanocomposites exhibit excellent thermal stability, abrasion resistance, thermal conductivity, and low heat accumulation and air permeability. It offers broad application prospects for the development of “green tires”. In the future, with further research, graphene/SBR composites are expected to be industrialized.

Overall, carbon-based fillers exhibit excellent mechanical, thermal, and optoelectronic properties. A key advantage of carbon-based fillers over other fillers is that they significantly improve thermal conductivity at lower loads. Therefore, rubber nanocomposites with carbon-based fillers have the potential to be used in specific thermal management applications. However, carbon-based fillers face multiple challenges for practical industrial applications in heat sink devices, including processing difficulties, poor electrical insulation, and cost issues [94]. Carbon-based thermally conductive fillers typically exhibit an anisotropic structure, which can lead to severe defects when used in tires under complex, harsh conditions. In addition, poor dispersion and low compatibility between the filler and the matrix may lead to spalling or delamination, reducing the durability and reliability of tires. To solve the problems of filler dispersion and interface compatibility, functionalization and compatibilization technologies are the key approaches. Through covalent modification or non-covalent modification, the surface energy of the filler can be effectively reduced, agglomeration can be inhibited, and the interfacial interaction between the filler and the rubber matrix can be enhanced, thereby improving the overall performance and durability of the composite material.

### 4.3. Ceramic Fillers

Usually, high-thermal-conductivity ceramic materials are divided into three main categories: oxides, nitrides, and carbides. Among them, nitrides and carbides have strong interatomic bonds, and their solid crystal structure significantly reduces phonon scattering [11], which effectively improves thermal conductivity. Next, the application of ceramic materials in tire rubber nanocomposites will be described in detail.

#### 4.3.1. Oxides

Oxide fillers are low-cost and offer significant advantages in practical applications. Therefore, research in thermally conductive rubber materials has received extensive attention. The commonly used material, Al_2_O_3_, is an ideal filler for industrial applications due to its good thermal conductivity, high resistivity, low cost, and non-toxicity. Among them, the most widely used form is α-Al_2_O_3_, a crystalline form with the highest stability. However, to achieve high thermal conductivity in rubber composites, high filler amounts (up to 70 wt%) are required, but this can come at the expense of mechanical properties, flexibility, and processability [95,96].

**Figure 10:**
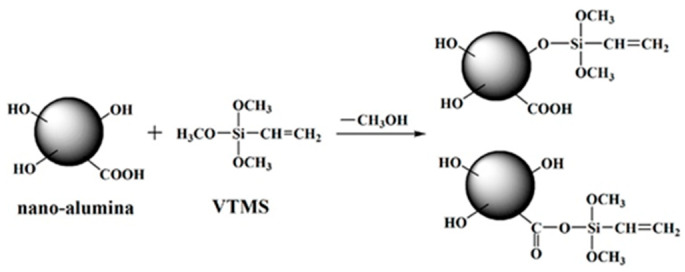
Schematic of in situ modification reactions between nano-alumina and VTMS during the curing procedure. Reprinted from [97], Copyright (2018), with permission from Elsevier.

Al_2_O_3_-filled rubber polymers inevitably face the challenge of thermal resistance. Therefore, researchers have conducted extensive studies on reducing thermal resistance. For Al_2_O_3_-based rubber composites, Al_2_O_3_ is usually modified using coupling agents. He et al. [97] used vinyl trimethoxysilane (VTMS) to modify Al_2_O_3_ to improve the compatibility between silicone rubber and Al_2_O_3_. Figure 10 illustrates the reaction mechanism for the process, showing that VTMS undergoes a condensation reaction with hydroxyl or carboxyl groups on the surface of alumina nanoparticles. Meanwhile, the introduction of VTMS led to the formation of covalent bonds between the filler and the rubber chains, thus firmly binding the filler to the matrix and improving the interfacial interactions. Compared with the pure matrix, the modified composites showed better dispersion and improved thermal conductivity by 104.7%, while the thermal conductivity of the SIR/alumina composites without VTMS increased by only 76.8%.

**Figure 11:**
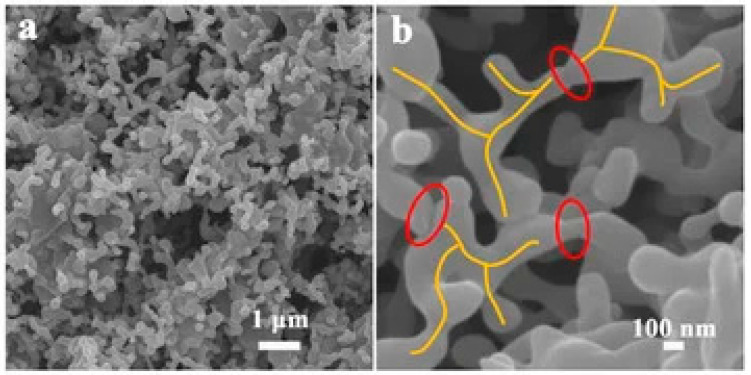
(**a**,**b**) B-Al_2_O_3_ SEM images. Adapted with permission from Ref. [98].

To address the damage caused by high loads on the mechanical properties of materials. Ouyang et al. [98] prepared silicone rubber composites using branched aluminum oxides (B-Al_2_O_3_). Scanning electron microscopy (SEM) revealed that the irregular branched chain structure promoted the overlapping distribution of fillers. The two-dimensional particles exhibited good dispersion. The formation of sintered necks reduced the interfacial thermal resistance between the filler and the matrix (Figure 11a,b). These properties enabled the material not only to enhance thermal conductivity but also to maintain excellent mechanical and electrical insulation.

ZnO is a semiconductor material widely used in sunscreens [99], sensors [100], biomedicine [101], photocatalysis [102], and the rubber industry. Meanwhile, as an essential vulcanization activator, ZnO can accelerate the vulcanization rate of rubber materials and improve their processing and physical properties. Most of the industrially produced ZnO is used in the manufacture of rubber products, especially tire products [103].

As an indispensable additive in the tire industry, zinc oxide can improve the thermal conductivity of tire rubber to a certain extent, which in turn promotes the dissipation of heat from the tire; however, the literature shows that there are still fewer applications of enhancing thermal conductivity through single zinc oxide particles filled with tire rubber composites, and more often this is achieved through hybridization.

In conclusion, oxide ceramic fillers, due to their low cost and easy availability, show great promise for rubber composites, especially in tires and other products. However, the poor thermal conductivity of oxide ceramic fillers and the need for high loading to fill the rubber matrix are their major drawbacks. Therefore, this problem can be solved by mixing with other anisotropic thermally conductive fillers.

#### 4.3.2. Nitrides

Nitride fillers are popular in heat dissipation due to their excellent thermal conductivity. Common nitride fillers include boron nitride, aluminum nitride, and silicon nitride.

**Figure 12:**
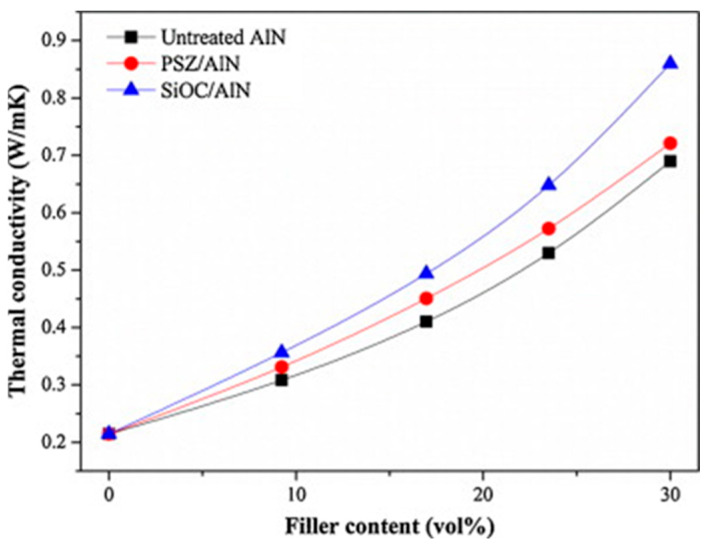
Thermal conductivity of silicone rubber filled untreated AlN, PSZ/AlN and SiOC/AlN at various filler content. Reprinted from [106], Copyright (2013), with permission from Elsevier.

Aluminum nitride (AlN) has high thermal conductivity, sound electrical insulation, low coefficient of thermal expansion, and high hardness [104,105]. However, aluminum nitride is prone to hygroscopicity and to hydrolysis, which can produce aluminum hydroxide with poor thermal conductivity, blocking the formation of thermal conduction channels. In industrial applications, aluminum nitride particles are often modified to improve their hydrolysis resistance. Aluminum nitride (AlN) coated with polysilazane (PSZ) and amorphous ceramic (SiOC) was prepared by dip-coating method. In addition, the PSZ/AlN and SiOC/AlN composites were embedded in a silicone rubber matrix. As shown in Figure 12, the thermal conductivity of the modified and treated PSZ/AlN and SiOC/AlN rubber composites was significantly higher than that of the untreated samples [106].

Despite its excellent thermal conductivity, aluminum nitride has been used less often in rubber composites, especially in mass-produced parts such as tires, due to its high cost, processing difficulty, and difficulty in matching properties.

Boron nitride is an ideal filler for high-thermal-conductivity rubbers. In addition to its inherent high thermal conductivity, high electrical resistivity, and low dielectric constant, boron nitride offers lubricity, low density, and excellent thermal stability [107,108]. Cubic boron nitride (c-BN) and hexagonal boron nitride (h-BN) are the two common crystal types. Among them, hexagonal boron nitride, also known as “white graphene” because of its graphite-like structure, has the best overall performance [109] and thus attracts much attention from researchers.

Given h-BN’s stable properties, its incorporation into matrices such as natural rubber [110], silicone rubber [111], nitrile rubber [112], and fluororubber [113] can significantly enhance thermal conductivity and enable thermal management of h-BN rubber composites under various working conditions.

When h-BN is used to fill the rubber matrix, problems such as poor dispersion, agglomeration, insignificant improvement of thermal conductivity, and degradation of mechanical properties often occur. To address these issues, a common approach is to modify the h-BN surface. At present, the modification of h-BN is mainly divided into non-covalent modification by physical force and covalent modification by chemical reaction.

**Figure 13:**
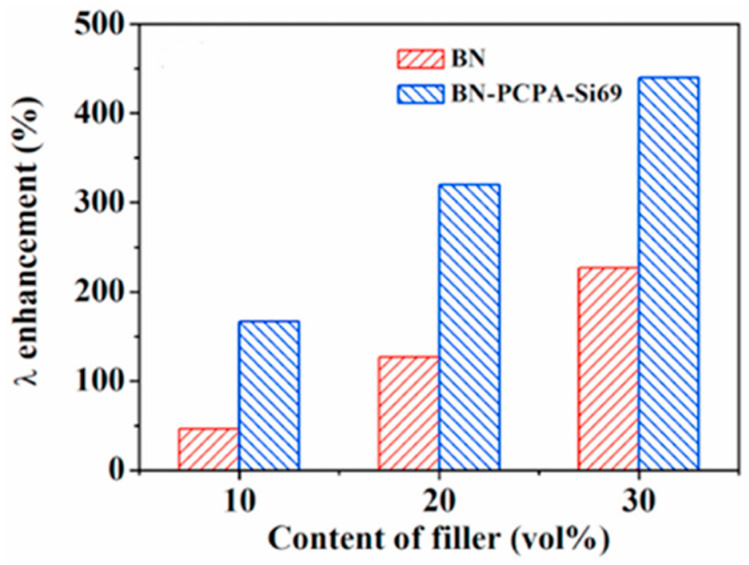
Enhancement in thermal conductivities of BN/NR and BN-PCPA-Si69/NR composites. Adapted with permission from Ref. [116]:Copyright (2020) Elsevier.

Yang et al. [114] researchers prepared BN-PDA-KH570/NR nanocomposites with excellent mechanical properties and high thermal conductivity using a combination of covalent and noncovalent methods. The specific experimental steps were as follows: h-BN was first modified with polydopamine (PDA), then grafted with a silane coupling agent (KH570), and finally, the treated BN and BN-PDA-KH570 flakes were doped into the NR matrix. Considering the high cost of polydopamine, researchers developed a low-cost poly(catechol/polyamine) (PCPA) layer to replace PDA [115]. In a follow-up experiment, Yang et al. [116] prepared BN-PCPA-Si69/NR composites by modifying BN flakes with PCPA and grafting them with Si69. Compared with pure NR, the thermal conductivity of this composite was substantially increased (Figure 13), and the mechanical properties were significantly improved.

BNNSs have been initially applied in polymer materials to improve thermal conductivity, especially in electronics. Their application in tire rubber composites is still immature, as large-scale production remains challenging.

**Figure 14:**
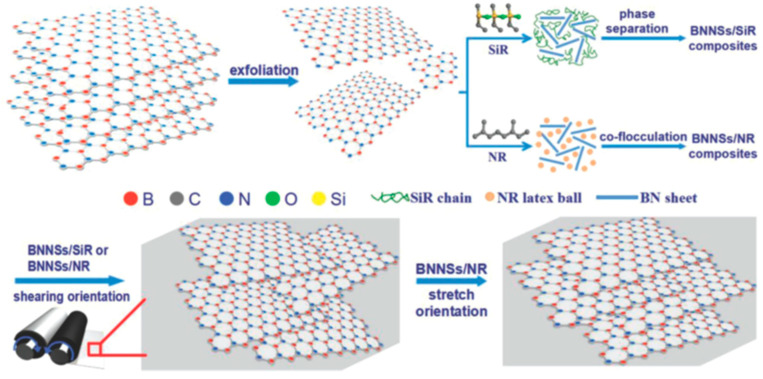
Fabrication of BNNSs/SiR and BNNSs/NR nanocomposites. Reprinted from [117]. Copyright (2014), with permission from John Wiley and Sons.

Kuang et al. [117] prepared a mixture of stripped BNNSs with isopropyl alcohol (IPA) via ultrasonication, diluted the solution with water, added natural rubber latex, and finally coagulated the mix with a 1.0 wt% aqueous solution of calcium chloride, yielding BNNSs/NR composites. The mixture required intense shear treatment to ensure uniform dispersion and directional alignment within the rubber matrix. Experimental results demonstrate that when the BNNS content reaches 24.0 vol%, the thermal conductivity of the BNNSs/NR composites is 10 times that of the pure NR matrix. The tire industry offers significant potential for the use of oriented, thermally conductive BNNSs in natural rubber composites (Figure 14).

**Figure 15:**
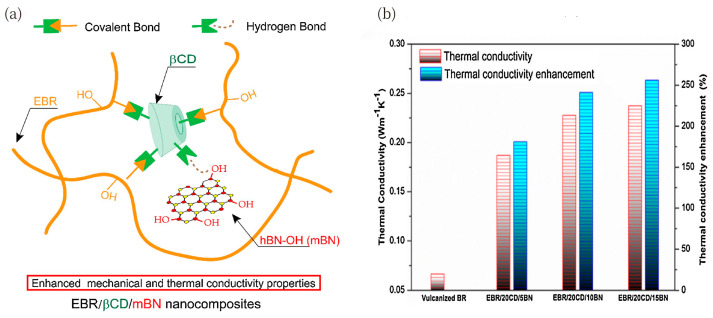
(**a**) Fabrication of EBR/βCD/mBN nanocomposites. (**b**) Trends in thermal conductivity of vulcanized BR and BR-based nanocomposites with different mBN loadings at 25 °C. Adapted with permission from Ref. [118]: Copyright (2019) Elsevier.

Poor dispersion and weak interfacial interactions are common problems of h-BN in a rubber matrix. To solve these problems, Yang et al. [118] proposed the use of biological β-cyclodextrin (βCD) as an interfacial cross-linking agent to promote the homogeneous dispersion of hydroxylated h-BN (mBN) in epoxy butadiene rubber (EBR) through hydrogen bonding to prepare EBR/CD/mBN nanocomposites, as shown in Figure 15a. The prepared composites showed enhanced interfacial interaction and significantly improved mechanical properties. Only 4 wt% mBN can dramatically improve thermal conductivity, indicating that h-BN has potential applications in tire tread rubber composites (Figure 15b).

Silicon nitride (Si_3_N_4_) is a critical thermally conductive and insulating ceramic material due to its low coefficient of thermal expansion, excellent high-temperature oxidation resistance, outstanding corrosion resistance, high fracture toughness, and high compressive and flexural strength [119,120]. Current studies on Si_3_N_4_-filled polymers to improve thermomechanical properties are mainly focused on various types of resin-based composites [121,122], whereas its application in a rubber matrix is relatively underexplored. Recent studies have used Si_3_N_4_ as a high-thermal-conductivity filler in SBR/BR matrix composites for the tire industry. The mechanical properties, including tensile and compressive, of the composites were significantly improved, especially at 6 phr filler content. In addition, the crosslinking density increased, the curing reaction accelerated, the scorch time shortened, and the thermal conductivity enhanced [123]. Due to the high cost, they are usually mixed with other lower-cost thermally conductive fillers.

In summary, nitride fillers, including aluminum nitride, boron nitride, and silicon nitride, are among the ideal choices for filling rubber-based polymers due to their excellent intrinsic thermal conductivity. However, their dispersion in rubber-based polymers, material compatibility, high cost, and processing difficulties have limited their development to the laboratory stage, and their application in large-scale products, such as tires, remains challenging.

#### 4.3.3. Carbides

Common carbide fillers include boron carbide (B_4_C) and silicon carbide (SiC).

Silicon carbide (SiC) is a compound formed through covalent bonding between carbon and silicon [124]. It exhibits high mechanical strength, a low coefficient of thermal expansion, robust chemical stability, and exceptional thermal conductivity among its numerous qualities [125]. As a desirable material for high-thermal-conductivity composites, SiC is widely used across various fields, including tire manufacturing. Studies indicate that the heat transfer characteristics of tires are significantly affected by their curing time and heat diffusion coefficient.

The effect of nano-SiC-filled styrene butadiene rubber/butadiene rubber (SBR/BR) on tire tread dynamics was investigated. According to the findings, SiC particles significantly accelerated the curing reaction and reduced the rubber matrix’s scorch time by about 10%. The results for the heat-diffusion coefficient showed that the addition of SiC increased the heat transfer rate during vulcanization by about 30%. The heat transfer rate after curing increased by about 50%, especially at a filler content of 5 phr, and the results of the de-heating data fitting showed that the tire heat-diffusion coefficient improved by about 97%, effectively preventing the tires from exploding at high temperatures [126].

After the introduction of B_4_C as a thermally conductive filler into natural rubber, the composite’s thermal conductivity improves, and its heat generation is significantly reduced. When the B_4_C filler is 45 vol%, the λ value is 0.76 W/m ·K [127].

Carbide ceramic fillers are prone to form anisotropic structures that form efficient thermal-conductivity networks and provide additional heat-transfer paths. However, by-products (carbon and graphite) from the synthesis process are difficult to remove, while the increased conductivity may be helpful in applications that require insulating properties.

### 4.4. Hybridized Fillers

Although the introduction of a single particle can enhance the thermal conductivity of composites, it may lead to a balance issue among dispersion, compatibility, and mechanical properties. Composite filling with fillers of different types, morphologies, and sizes not only enhances compactness and reduces viscosity, but also effectively creates synergistic effects on the thermal conductivity channels [128].

The application of hybridized fillers in rubber matrix composites has become a new trend. Table 2 provides a detailed summary of recent examples of hybridized fillers filled into a rubber matrix to enhance thermal conductivity [129,130,131,132,133,134,135,136,137,138,139,140,141,142]. In the tire industry, the introduction of thermally conductive hybrid fillers provides a new solution for large-scale formulations and is expected to drive industry advancements in several areas.

**Figure 16:**
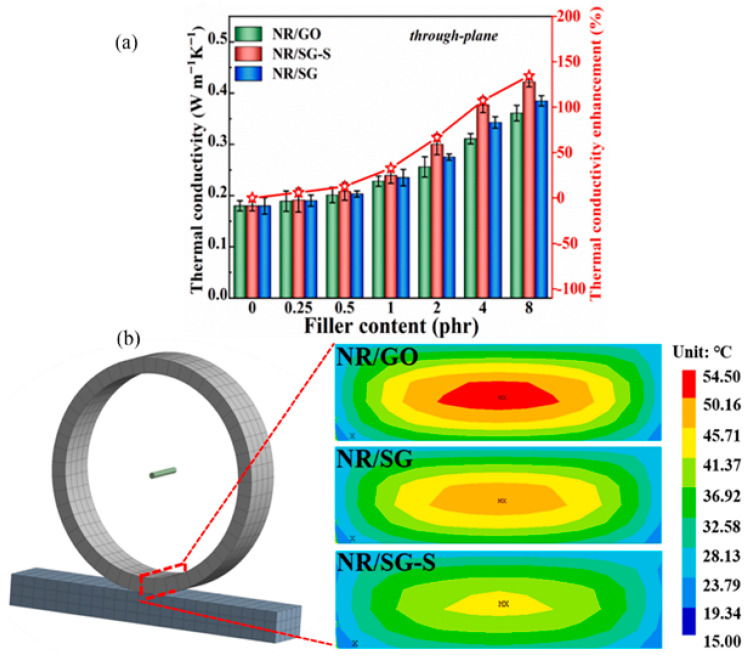
(**a**) Thermal conductivity of NR/GO, NR/SG, and NR/SG-S composites with different filler loadings, (**b**) Temperature field of the simulated rubber tires of NR/GO, NR/SG, and NR/SG-S by using ANSYS finite element. Adapted with permission from Ref. [143]: Copyright (2022) Elsevier.

Currently, preparing graphene-rubber composites that combine high thermal conductivity, high mechanical properties, and low heat accumulation remains challenging. Duan et al. [143] prepared SiC/GO(SG) fillers by hybridizing GO with SiC. Subsequently, SG-S multifunctional particles were formed by chemical deposition of nano-sulfur on their surfaces, and finally, NR/SG-S composites were prepared. Notably, the SG-S filler not only improved the interfacial interaction between the filler and the rubber but also achieved a uniform dispersion of the vulcanizing agent. The results demonstrated that the thermal conductivity was enhanced by 21.2% when the SG-S filler was doped at 4 phr compared to NR/GO (Figure 16a), thereby significantly improving the tensile strength of the composites and reducing heat buildup. In addition, simulation results show that NR/SG-S tires have better thermal control than NR/GO tires (Figure 16b), with a maximum temperature 8 °C lower, which helps to mitigate thermal damage and extend service life.

Previous studies have shown a synergistic interaction between carbon nanotubes and carbon black. Blended fillers of carbon nanotubes and carbon black have shown promise for reducing low-heat buildup in NR vulcanized rubber [49,144].

For example, by improving the dispersion state of MWCNT and enabling its blending with CB, the focus was on the effect on the heat accumulation properties of NR/SBR-based tire tread rubber. Analytical findings indicated that the addition of nano-MWCNT significantly enhanced the composites’ thermal conductivity and reduced heat accumulation, thereby extending tire service life. Meanwhile, curing parameters such as abrasion resistance and tensile strength were also significantly improved [145].

In addition to the above-mentioned system, constructing an efficient thermal conduction network by leveraging the interaction among fillers of different dimensions is another important strategy to give full play to the synergistic advantages of hybrid fillers. Wang et al. [146] reported a PCL-SiO_2_/SBR composite material. The synergistic enhancement mechanism lies in the fact that the hydrogen bonds and electrostatic interactions between PKL and SiO_2_ effectively inhibit the agglomeration of fillers, ensuring their uniform dispersion in rubber. Meanwhile, the two work in synergy to enhance the interaction with the rubber chain, constructing a solid filler network. Compared with carbon black, this structure endows the material with superior mechanical properties, as well as lower rolling resistance and heat generation. Similarly, another study by Wang et al. [147] demonstrated that combining two-dimensional graphene with zero-dimensional carbon black, silicon dioxide, and one-dimensional carbon nanotubes, respectively, can form a more efficient heat conduction network. Specifically, graphene and carbon black can mutually inhibit agglomeration and reduce interfacial thermal resistance. Silicon dioxide and graphene form a “point-surface” structure, enhancing the transmission path of phonons. The combination of graphene and carbon nanotubes achieves 1D-2D synergy, jointly constructing three-dimensional heat conduction channels. These synergistic strategies based on different mechanisms of action not only enhance the wear resistance of rubber composites but also significantly improve their thermal conductivity, further highlighting the huge potential of hybrid fillers in tire heat dissipation applications.

**Figure 17:**
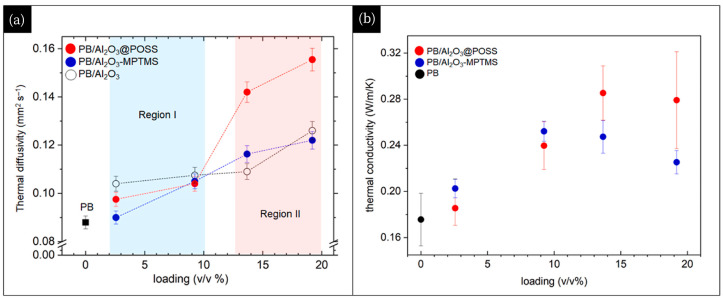
Trends of thermal diffusivity (α) and thermal conductivity (k) for homopolymerized PB and PB filled with unmodified and functionalized Al_2_O_3_ NPs. Reprinted from [148], Copyright (2023), with permission from Elsevier.

In exploring the application of Al_2_O_3_ in tire rubber composites, a novel filler system was developed. By introducing polyhedral siloxane (POSS) units, Al_2_O_3_@POSS hybrid fillers were formed. The presence of POSS units was shown to be crucial for forming continuous networks and achieving compatibility between fillers and polymers. Notably, at low filler loadings (15–10 *v*/*v*%), this modified filler exhibited a remarkable enhancement in thermal diffusivity as well as an improvement in thermal conductivity (Figure 17). This study lays the foundation for the large-scale application of Al_2_O_3_@POSS hybrid filler in tire rubber composites [148].

In the face of global energy constraints and environmental challenges, the tire industry has proposed “green tires” as a positive response. On the one hand, we are concerned about the impact of tire performance on vehicle performance and economic benefits; on the other hand, we are also worried about the environmental issues associated with tire waste. Therefore, sustainable bio-based fillers are becoming a popular choice for rubber composites [149].

Lignin, a renewable natural polymer, has been successfully incorporated into a rubber matrix and has demonstrated unique advantages in curing, dynamic mechanical, and thermal properties [150]. Butadiene rubber composites containing graphene oxide and lignin fillers were prepared using lignin as a raw material to achieve their biodegradability. The results showed that the lignin-filled composites have excellent heat resistance and are suitable for extreme weather conditions. In addition, the 1 wt% lignin-filled SBR exhibited good morphological characteristics and high porosity, along with exceptional mechanical and biodegradability properties, making it suitable for tire applications. Therefore, 1 wt% lignin-filled SBR composites are considered a potential choice for future green tire applications [151].

In summary, hybridized fillers excel at thermal management in rubber composites due to their synergistic effects. A variety of hybridized fillers can be effectively combined with the rubber matrix to maintain the rubber’s processability and moldability. In hybrid materials, the ratio of the different materials significantly affects the network structure and the orientation of the fillers, and thus the homogeneity of heat transfer from the matrix. This feature is crucial for rubber nanocomposites in tires. In tire design, two aspects must be balanced to extend product life. One is to enhance the tire’s heat dissipation capacity to prevent performance degradation at high temperatures, and the other is to avoid ply delamination or spalling during use. This balance is crucial for the durability and safety of tires [152].

## 5. Application of Microsimulation in Optimizing Thermal Conductivity of Rubber Tires

With the synergistic development of multiple disciplines, the importance of simulation methods in scientific research is increasing. Simulation, theory, and experiment complement and verify each other.

Tire temperature is a key factor affecting its durability and performance, and the failure characteristics of tire rubber are closely related to temperature. The method of finite element analysis combined with experiment has been used for decades to study the internal temperature distribution in tires [153,154]. This combination not only improves the reliability of predictions but also advances tire technology.

In recent years, molecular dynamics (MD) simulations have become an essential tool for scientific research, providing detailed microstructural and kinetic data that reveal material properties [155]. Traditional tire heat dissipation studies have focused on macroscopic composite formulation design and thermodynamic analysis. In contrast, molecular dynamics simulations can elucidate the microscopic mechanism of heat transport in rubber composites.

The impact of the rubber matrix structure on heat conductivity can be examined using simulation techniques. For example, Zhao et al. [156] investigated the effects of styrene composition ratio, temperature, and tensile strain on the thermal conductivity of SBR using inverse nonequilibrium molecular dynamics simulations with full atomic resolution. The findings showed that the thermal conductivity of SBR decreased as the styrene content increased. In the direction of elongation, tensile strain exhibited an augmentation in thermal conductivity; however, in the perpendicular orientation, it demonstrated a reduction. With increasing temperature, thermal conductivity first increases and then decreases. This phenomenon stems from changes in phonon transport modes. The results of the study are essential for the design of SBR thermal conductivity.

Molecular dynamics simulations can comprehensively elucidate the interactions between fillers and the matrix, thereby facilitating the rational design of filler-reinforced rubbers. For instance, Yang et al. [157] integrated molecular dynamics simulations with experimental methods to investigate how the degree of oxidation of graphene oxide (GO) influences dispersion, interfacial adhesion, and heat transfer in butadiene-styrene-vinyl pyridine rubber (VPR)/GO composites. The findings demonstrated that at 15% oxidation, GO dispersion, composite mechanical characteristics, and thermal conductivity were all optimized. Additionally, to thoroughly investigate how GO differs from other rubber matrices in terms of interfacial interactions. Fu et al. [158] built a composite model via MD simulation and combined it with experiments to explore the enhancement mechanism of GO on NR, SBR, and XNBR. The results indicated that graphene oxide (GO) exhibited optimal dispersion in XNBR, whereas its dispersion in natural rubber (NR) was suboptimal, failing to enhance the material’s properties significantly. This study offers a novel approach for the design of high-performance rubber nanocomposites.

The pretreatment of fillers with specific surface modification strategies has been proven to be the key to optimizing the performance of composite materials. This point was fully demonstrated in the boron nitride-filled system that was deeply explored through molecular dynamics simulation. In this study, through a simple non-covalent modification process, boron nitride (BN) was treated with superdispersants, effectively improving the thermal conductivity and tensile properties of natural rubber (NR) composites while maintaining their intrinsic structure. The results of molecular dynamics simulations indicate that this enhancement effect stems from the increased molecular binding energy and the reduced free volume fraction after the introduction of the dispersant, which confirms the improvement of interfacial compatibility at the microscopic level [159]. Yang et al. [160] explored the enhancing effect and microscopic mechanism of heat transfer at the interface of composite materials after hexagonal boron nitride (h-BN) was modified by both covalent and non-covalent methods. The research combined ACA ball milling with TA self-assembly to achieve the functionalization of h-BN. The simulation results show that the dual modification can significantly reduce the interfacial thermal resistance and increase the thermal conductivity, while the thermal stability of the composite material is also improved simultaneously. This synergy effect is attributed to the combined effect of the strengthening of interface bonding and the optimization of phonon vibration power spectra. It is of great significance for understanding the heat conduction mechanism and material design of filled thermal interface materials.

In addition, Liang et al. [161] explored the use of molecular dynamics-based CAPD models in designing tire rubber polymer products with good heat dissipation properties. Taking the butadiene rubber structure as an example, the calibration by the MD method revealed that the properties of polybutadiene, such as thermal conductivity and density, were found to be in good agreement with the reference data, which verified the validity of MD in property model development.

In conclusion, molecular dynamics simulations provide a new perspective to study the microstructure of rubber composites and their heat transfer mechanisms. With the continuous advancement of computational technology, molecular dynamics simulation will be able to handle more complex material systems and is expected to provide stronger theoretical support for the interface modification of rubber nanocomposites. As research deepens, we expect more breakthroughs and advances in the field of rubber materials in the future.

## 6. Summary

With continuous advances in materials science and growing market demand, thermally conductive rubber composites are anticipated to find applications across an increasingly diverse range of fields. This research systematically investigates the mechanisms underlying the thermal conductivity of rubber-matrix composites and the factors that influence it. A comprehensive analysis of the thermal challenges associated with tire rubber, with special emphasis on the use of various thermally conductive fillers to improve heat dissipation in tire formulations. It aims to provide crucial theoretical support and a data basis for the formulation design of tire rubber through molecular dynamics simulation, focusing on the heat conduction mechanism, filler influence, and optimization of the crosslinking structure.

This paper primarily discusses the thermal-conductivity mechanism of rubber and its polymers from the perspective of fillers, focusing on the effect of fillers on thermal conductivity. Still, it does not delve into the impact of the rubber matrix structure on thermal conductivity. Although the theoretical modeling of thermally conductive rubber is not explored in this paper, this area has always been a hot research topic. Current thermal conductivity models are usually specialized and poorly generalized and cannot effectively predict the thermal conductivity of various systems constructed from new materials. Therefore, a universal thermal conductivity model applicable to a variety of fillers will be the focus of future research.

Although rubber composites show great potential for enhancing thermal conductivity and reducing heat buildup in tires, there are still many problems with current research, including (1) high cost and processing difficulties make tires difficult to produce on a large scale, leading to slow progress in industrialization; (2) uniform dispersion of heat-conducting particles in the rubber matrix is still a challenge; and (3) a high content of fillers may lead to a degradation of mechanical properties.

Based on the above problems, future research will focus on the following aspects: First, optimize processing technology and use electric and magnetic fields, as well as 3D printing, to improve the compatibility and orientation of fillers in the rubber matrix. Second, actively explore emerging nanofillers, such as nanocomposites. Third, to build a multifunctional hybridization system for fillers. Fourthly, an in-depth study of the chemical properties of materials and the thermal changes during thermal conductivity, seeking efficient surface treatment methods. We believe that, in the near future, the performance of thermally conductive rubber composites will continue to improve, thereby providing a stronger guarantee of tire safety and durability.

## Figures and Tables

**Table 1:** Values of common thermally conductive fillers.

Fillers	Thermal Conductivity (W/m·K)	Ref
Silver	450	[41]
Copper	483	[41]
Aluminum	204	[41]
Nickel	158	[41]
Gold	345	[41]
Zinc	121	[26]
Iron	80	[26]
Zinc oxide	30	[26]
Beryllium oxide	270	[42,43]
Aluminum oxide	20–29	[41]
Aluminum nitride	320	[26]
Silicon nitride	>150	[44]
Silicon carbide	80	[26]
Boron nitride	250–300	[41]
BN nanosheets	751 (λ_||_) *	[45]
Carbon black	6–174	[41]
Carbon Nanotubes	2000–6000	[41]
Carbon fiber	1200	[46]
Diamond	2000	[41]
Graphite	100–400 (λ_||_)	[41]
Graphene	600–2800 (λ_||_)	[47]
Molybdenum sulfide	34.5	[48]

* λ_||_ represents the in-plane thermal conductivity.

**Table 2:** Recent examples of hybrid fillers filled with different rubber matrixes.

Hybrid Fillers	Rubber Matrix	Filler Loading	Filler Type	λ_1_	λ_2_	Ref.
PCNT@RGO	SBR	3 wt%	1D+2D	0.23	0.45	[129]
Al_2_O_3_@TA-Fe^3+^@Ag	NBR	50 vol%		0.15	0.90	[130]
Al_2_O_3_-PRd@BN-PRd	ENR	30 vol%	0D+2D	0.1390	0.5147	[131]
rGO-PDA@ Al_2_O_3_	NR	25 vol%	0D+2D	0.1726	0.863	[132]
SiCNWs@rGO	SR	1.84 vol%	1D+2D	1.66	2.74	[133]
Al_2_O_3_-PDA@Ag	NR	10 vol%		0.10	0.20	[134]
CF@ Al_2_O_3_	SR	25 vol%	0D+1D	-	9.6	[135]
Zn@ZnO@CF	SR	60 phr		0.48	1.53	[136]
rGO@ Al_2_O_3_	NR	18 vol%	0D+2D	0.168	0.514	[137]
BN@rGO	NR	4.9 vol%		0.18	1.28 (λ_||_)	[138]
Al_2_O_3_@PCPA@GO	XNBR	30 vol%	0D+2D	0.16	0.48	[139]
AlN@CNT(1:1)	NR	12 vol%	1D+2D	-	0.502	[140]
CF@BN	SR	CF30 vol%BN10 vol%	1D+2D	-	2.789	[141]
T-rGO@BN	PDMS	14.3 vol%		-	1.41	[142]

## Data Availability

No new data were created or analyzed in this study. Data sharing is not applicable to this article.

## References

[1] Li Z., Sun Y., Hu F., Liu D., Zhang X., Ren J., Guo H., Shalash M., He M., Hou H. (2025). An Overview of Polymer-Based Thermally Conductive Functional Materials. J. Mater. Sci. Technol..

[2] Niu H., Cao Y., Yang K., Dong H., Cheng Q., Chen Y. (2025). Review of Boron Nitride Based Polymer Composites with Ultrahigh Thermal Conductivity: Critical Strategies and Applications. Compos. Part A Appl. Sci. Manuf..

[3] Li Q., Ding J., Zhang Y., Lei W., Wei Z., Chen C., Shi D. (2024). Discussion on the Thermal Conductive Network Threshold of Al_2_O_3_/Co-Continuous Phase Polymer Composites. Nano Mater. Sci..

[4] Ruan K., Guo Y., Gu J. (2021). Liquid Crystalline Polyimide Films with High Intrinsic Thermal Conductivities and Robust Toughness. Macromolecules.

[5] Khan A., Puttegowda M., Jagadeesh P., Marwani H.M., Asiri A.M., Manikandan A., Parwaz Khan A.A., Ashraf G.M., Rangappa S.M., Siengchin S. (2022). Review on Nitride Compounds and Its Polymer Composites: A Multifunctional Material. J. Mater. Res. Technol..

[6] Danilova-Tret′yak S.M. (2016). On Thermophysical Properties of Rubbers and Their Components. J. Eng. Phys. Thermophys..

[7] Salkhi Khasraghi S., Momenilandi M., Shojaei A. (2022). Tire Tread Performance of Silica-Filled SBR/BR Rubber Composites Incorporated with Nanodiamond and Nanodiamond/Nano-SiO_2_ Hybrid Nanoparticle. Diam. Relat. Mater..

[8] Mazumder M.R.H., Mathews L.D., Mateti S., Salim N.V., Parameswaranpillai J., Govindaraj P., Hameed N. (2022). Boron Nitride Based Polymer Nanocomposites for Heat Dissipation and Thermal Management Applications. Appl. Mater. Today.

[9] Burger N., Laachachi A., Ferriol M., Lutz M., Toniazzo V., Ruch D. (2016). Review of Thermal Conductivity in Composites: Mechanisms, Parameters and Theory. Prog. Polym. Sci..

[10] Chen Q., Yang K., Feng Y., Liang L., Chi M., Zhang Z., Chen X. (2024). Recent Advances in Thermal-Conductive Insulating Polymer Composites with Various Fillers. Compos. Part A Appl. Sci. Manuf..

[11] Chen H., Ginzburg V.V., Yang J., Yang Y., Liu W., Huang Y., Du L., Chen B. (2016). Thermal Conductivity of Polymer-Based Composites: Fundamentals and Applications. Prog. Polym. Sci..

[12] Kim S.J., Hong C., Jang K.-S. (2019). Theoretical Analysis and Development of Thermally Conductive Polymer Composites. Polymer.

[13] Wang T., Lin Y., Li P., Jiang P., Zhang C., Xu H., Xie H., Huang X. (2022). Unidirectional Thermal Conduction in Electrically Insulating Phase Change Composites for Superior Power Output of Thermoelectric Generators. Compos. Sci. Technol..

[14] Yang J., Shen X., Yang W., Kim J. (2023). Templating Strategies for 3D-Structured Thermally Conductive Composites: Recent Advances and Thermal Energy Applications. Prog. Mater. Sci..

[15] Li A., Zhang C., Zhang Y.-F. (2017). Thermal Conductivity of Graphene-Polymer Composites: Mechanisms, Properties, and Applications. Polymers.

[16] Soga K., Saito T., Kawaguchi T., Satoh I. (2017). Percolation Effect on Thermal Conductivity of Filler-Dispersed Polymer Composites. J. Therm. Sci. Technol..

[17] Zhang X., Wu K., Liu Y., Yu B., Zhang Q., Chen F., Fu Q. (2019). Preparation of Highly Thermally Conductive but Electrically Insulating Composites by Constructing a Segregated Double Network in Polymer Composites. Compos. Sci. Technol..

[18] Xu X., Chen J., Zhou J., Li B. (2018). Thermal Conductivity of Polymers and Their Nanocomposites. Adv. Mater..

[19] Guo Y., Qiu H., Ruan K., Wang S., Zhang Y., Gu J. (2022). Flexible and Insulating Silicone Rubber Composites with Sandwich Structure for Thermal Management and Electromagnetic Interference Shielding. Compos. Sci. Technol..

[20] Suh D., Moon C.M., Kim D., Baik S. (2016). Ultrahigh Thermal Conductivity of Interface Materials by Silver-Functionalized Carbon Nanotube Phonon Conduits. Adv. Mater..

[21] Gu J., Xie C., Li H., Dang J., Geng W., Zhang Q. (2014). Thermal Percolation Behavior of Graphene Nanoplatelets/Polyphenylene Sulfide Thermal Conductivity Composites. Polym. Compos..

[22] Oluwalowo A., Nguyen N., Zhang S., Park J.G., Liang R. (2019). Electrical and Thermal Conductivity Improvement of Carbon Nanotube and Silver Composites. Carbon.

[23] Aradhana R., Mohanty S., Nayak S.K. (2019). Novel Electrically Conductive Epoxy/Reduced Graphite Oxide/Silica Hollow Microspheres Adhesives with Enhanced Lap Shear Strength and Thermal Conductivity. Compos. Sci. Technol..

[24] Su Y., Li J.J., Weng G.J. (2018). Theory of Thermal Conductivity of Graphene-Polymer Nanocomposites with Interfacial Kapitza Resistance and Graphene-Graphene Contact Resistance. Carbon.

[25] Guo Y., Ruan K., Wang G., Gu J. (2023). Advances and Mechanisms in Polymer Composites toward Thermal Conduction and Electromagnetic Wave Absorption. Sci. Bull..

[26] Guo Y., Ruan K., Shi X., Yang X., Gu J. (2020). Factors Affecting Thermal Conductivities of the Polymers and Polymer Composites: A Review. Compos. Sci. Technol..

[27] Zhi J., Wang S., Zhang M., Wang H., Lu H., Lin W., Qiao C., Hu C., Jia Y. (2019). Numerical Analysis of the Dependence of Rubber Hysteresis Loss and Heat Generation on Temperature and Frequency. Mech. Time-Depend. Mater..

[28] Liu Y., Chen W., Jiang D. (2022). Review on Heat Generation of Rubber Composites. Polymers.

[29] Lin Y.-J., Hwang S.-J. (2004). Temperature Prediction of Rolling Tires by Computer Simulation. Math. Comput. Simul..

[30] Bafrnec M., Juma M., Toman J., Jurĉiová J., Kuĉma A. (1999). Thermal Diffusivity of Rubber Compounds. Plast. Rubber Compos..

[31] Dong H., Zhang Y. (2023). Robust, Thermally Conductive and Damping Rubbers with Recyclable and Self-Healable Capability. Compos. Part A Appl. Sci. Manuf..

[32] Abeygunawardane G.A., Weragoda S., Senevirathne N., Liyanage E. (2024). Characterization of Curing Status of Commercial Tire Compounds with Vein Graphite Powder and Its Particle Sizes—Experimental and Computational Study. J. Appl. Polym. Sci..

[33] Wu J., An S., Teng F., Su B.L., Wang Y.S. (2024). Thermo-Mechanical Behavior of Solid Rubber Tire under High-Speed Free Rolling Conditions. Polym. Bull..

[34] Cattani P., Cattani L., Magrini A. (2023). Tyre–Road Heat Transfer Coefficient Equation Proposal. Appl. Sci..

[35] Gaska K., Rybak A., Kapusta C., Sekula R., Siwek A. (2015). Enhanced Thermal Conductivity of Epoxy–Matrix Composites with Hybrid Fillers. Polym. Adv. Technol..

[36] Li J., Liu X., Feng Y., Yin J. (2022). Recent Progress in Polymer/Two-Dimensional Nanosheets Composites with Novel Performances. Prog. Polym. Sci..

[37] Wei X., Luo T. (2019). Chain Length Effect on Thermal Transport in Amorphous Polymers and a Structure–Thermal Conductivity Relation. Phys. Chem. Chem. Phys..

[38] Boudenne A., Mamunya Y., Levchenko V., Garnier B., Lebedev E. (2015). Improvement of Thermal and Electrical Properties of Silicone–Ni Composites Using Magnetic Field. Eur. Polym. J..

[39] Zhao W., Zhang M., Li H., Du Y., Bu Q., Cao L., Zong C. (2022). Mussel Inspired Modification of Nanodiamonds for Thermally Conductive, and Electrically Insulating Rubber Composites. Diam. Relat. Mater..

[40] Shiva M., Kamkar Dallakeh M., Ahmadi M., Lakhi M. (2021). Effects of Silicon Carbide as a Heat Conductive Filler in Butyl Rubber for Bladder Tire Curing Applications. Mater. Today Commun..

[41] Han Z., Fina A. (2011). Thermal Conductivity of Carbon Nanotubes and Their Polymer Nanocomposites: A Review. Prog. Polym. Sci..

[42] Akishin G.P., Turnaev S.K., Vaispapir V.Y., Gorbunova M.A., Makurin Y.N., Kiiko V.S., Ivanovskii A.L. (2009). Thermal Conductivity of Beryllium Oxide Ceramic. Refract. Ind. Ceram..

[43] Wang X., Wang R., Peng C., Li T., Liu B. (2010). Synthesis and Sintering of Beryllium Oxide Nanoparticles. Prog. Nat. Sci. Mater. Int..

[44] Hirao K., Watari K., Hayashi H., Kitayama M. (2001). High Thermal Conductivity Silicon Nitride Ceramic. MRS Bull..

[45] Cai Q., Scullion D., Gan W., Falin A., Zhang S., Watanabe K., Taniguchi T., Chen Y., Santos E.J.G., Li L.H. (2019). High Thermal Conductivity of High-Quality Monolayer Boron Nitride and Its Thermal Expansion. Sci. Adv..

[46] Han S., Ji Y., Zhang Q., Wu H., Guo S., Qiu J., Zhang F. (2023). Tetris-Style Stacking Process to Tailor the Orientation of Carbon Fiber Scaffolds for Efficient Heat Dissipation. Nano-Micro Lett..

[47] Mohapatra A., Das S., Majumdar K., Ramachandra Rao M.S., Jaiswal M. (2021). Thermal Transport across Wrinkles in Few-Layer Graphene Stacks. Nanoscale Adv..

[48] Mehra N., Mu L., Ji T., Yang X., Kong J., Gu J., Zhu J. (2018). Thermal Transport in Polymeric Materials and across Composite Interfaces. Appl. Mater. Today.

[49] Yu S., Tang Z., Fang S., Wu S., Guo B. (2021). Polyrhodanine Mediated Interface in Natural Rubber/Carbon Black Composites toward Ultralow Energy Loss. Compos. Part A Appl. Sci. Manuf..

[50] Gao B.Z., Xu J.Z., Peng J.J., Kang F.Y., Du H.D., Li J., Chiang S.W., Xu C.J., Hu N., Ning X.S. (2015). Experimental and Theoretical Studies of Effective Thermal Conductivity of Composites Made of Silicone Rubber and Al2O3 Particles. Thermochim. Acta.

[51] Li Y.-T., Liu W.-J., Shen F.-X., Zhang G.-D., Gong L.-X., Zhao L., Song P., Gao J.-F., Tang L.-C. (2022). Processing, Thermal Conductivity and Flame Retardant Properties of Silicone Rubber Filled with Different Geometries of Thermally Conductive Fillers: A Comparative Study. Compos. Part B Eng..

[52] Zhou W., Qi S., Tu C., Zhao H., Wang C., Kou J. (2007). Effect of the Particle Size of Al_2_O_3_ on the Properties of Filled Heat-conductive Silicone Rubber. J. Appl. Polym. Sci..

[53] Zhou W., Qi S., Zhao H., Liu N. (2007). Thermally Conductive Silicone Rubber Reinforced with Boron Nitride Particle. Polym. Compos..

[54] He Y., Chen Z.C., Ma L.X. (2009). Thermal Conductivity and Mechanical Properties of Silicone Rubber Filled with Different Particle Sized SiC. Adv. Mater. Res..

[55] Suntako R. (2018). Effect of Synthesized ZnO Nanoparticles on Thermal Conductivity and Mechanical Properties of Natural Rubber. IOP Conf. Ser. Mater. Sci. Eng..

[56] Kemaloglu S., Ozkoc G., Aytac A. (2010). Properties of Thermally Conductive Micro and Nano Size Boron Nitride Reinforced Silicon Rubber Composites. Thermochim. Acta.

[57] Matsubara H., Ohara T. (2021). Effect of the In-Plane Aspect Ratio of a Graphene Filler on Anisotropic Heat Conduction in Paraffin/Graphene Composites. Phys. Chem. Chem. Phys..

[58] Evgin T., Koca H.D., Horny N., Turgut A., Tavman I.H., Chirtoc M., Omastová M., Novak I. (2016). Effect of Aspect Ratio on Thermal Conductivity of High Density Polyethylene/Multi-Walled Carbon Nanotubes Nanocomposites. Compos. Part A Appl. Sci. Manuf..

[59] Kong S.M., Mariatti M., Busfield J.J.C. (2011). Effects of Types of Fillers and Filler Loading on the Properties of Silicone Rubber Composites. J. Reinf. Plast. Compos..

[60] Aggarwal A., Hackel N., Grunert F., Ilisch S., Beiner M., Blume A. (2024). Investigation of Rheological, Mechanical, and Viscoelastic Properties of Silica-Filled SSBR and BR Model Compounds. Polymers.

[61] Liu Z., Liang C., Yan Y., Tao Y., An G., Li T. (2025). Effect of Noncovalent Bonding Modified Graphene on Thermal Conductivity of Graphene/Natural Rubber Composites Based on Molecular Dynamics Approach. Langmuir.

[62] Jia C., Zhang P., Seraji S.M., Xie R., Chen L., Liu D., Xiong Y., Chen H., Fu Y., Xu H. (2022). Effects of BN/GO on the Recyclable, Healable and Thermal Conductivity Properties of ENR/PLA Thermoplastic Vulcanizates. Compos. Part A Appl. Sci. Manuf..

[63] Jaberi Mofrad F., Ostad Movahed S., Ahmadpour A. (2024). Surface Modification of Commercial Carbon Black by Silane Coupling Agents for Improving Dispersibility in Rubber Compounds. J. Appl. Polym. Sci..

[64] Zhang X., Yi J., Yin Y., Song Y., Xiong C. (2021). Thermal Conductivity and Electrical Insulation Properties of H-BN@PDA/Silicone Rubber Composites. Diam. Relat. Mater..

[65] Feng W., Qin M., Feng Y. (2016). Toward Highly Thermally Conductive All-Carbon Composites: Structure Control. Carbon.

[66] Gao J., He Y., Gong X. (2018). Effect of Electric Field Induced Alignment and Dispersion of Functionalized Carbon Nanotubes on Properties of Natural Rubber. Results Phys..

[67] Ding D., Huang R., Wang X., Zhang S., Wu Y., Zhang X., Qin G., Liu Z., Zhang Q., Chen Y. (2022). Thermally Conductive Silicone Rubber Composites with Vertically Oriented Carbon Fibers: A New Perspective on the Heat Conduction Mechanism. Chem. Eng. J..

[68] Ji J., Chiang S.-W., Liu M., Liang X., Li J., Gan L., He Y., Li B., Kang F., Du H. (2020). Enhanced Thermal Conductivity of Alumina and Carbon Fibre Filled Composites by 3-D Printing. Thermochim. Acta.

[69] An D., Duan X., Cheng S., Zhang Z., Yang B., Lian Q., Li J., Sun Z., Liu Y., Wong C.-P. (2020). Enhanced Thermal Conductivity of Natural Rubber Based Thermal Interfacial Materials by Constructing Covalent Bonds and Three-Dimensional Networks. Compos. Part A Appl. Sci. Manuf..

[70] Wang Z., Fan L., Li R., Xu Y., Fu Q. (2022). Preparation of Polymer Composites with High Thermal Conductivity by Constructing a “Double Thermal Conductive Network” via Electrostatic Spinning. Compos. Commun..

[71] Zhang X., Cai L., He A., Ma H., Li Y., Hu Y., Zhang X., Liu L. (2021). Facile Strategies for Green Tire Tread with Enhanced Filler-Matrix Interfacial Interactions and Dynamic Mechanical Properties. Compos. Sci. Technol..

[72] Jia L.-C., Jin Y.-F., Ren J.-W., Zhao L.-H., Yan D.-X., Li Z.-M. (2021). Highly Thermally Conductive Liquid Metal-Based Composites with Superior Thermostability for Thermal Management. J. Mater. Chem. C.

[73] Liao P., Guo H., Niu H., Li R., Yin G., Kang L., Ren L., Lv R., Tian H., Liu S. (2024). Core–Shell Engineered Fillers Overcome the Electrical-Thermal Conductance Trade-Off. ACS Nano.

[74] Sun Z., Li J., Yu M., Kathaperumal M., Wong C.-P. (2022). A Review of the Thermal Conductivity of Silver-Epoxy Nanocomposites as Encapsulation Material for Packaging Applications. Chem. Eng. J..

[75] Zhang J., Kong Z., An Q., Wu T., Zou L. (2024). A Flexible Thermal Interface Composite of Copper-Coated Carbon Felts with 3d Architecture in Silicon Rubber. Polymer.

[76] Zhang A., Li Y. (2023). Thermal Conductivity of Aluminum Alloys—A Review. Materials.

[77] Niu H.J., Zhang Z.Y., Guo W., Xue Y., Yao Z.X. (2012). Mechanical, Morphological and Thermally Behaviors of Natural Rubber/Aluminum Powder Composites. Key Eng. Mater..

[78] Jang S., Choi E.J., Cheon H.J., Choi W.I., Shin W.S., Lim J.-M. (2021). Fabrication of Al_2_O_3_/ZnO and Al_2_O_3_/Cu Reinforced Silicone Rubber Composite Pads for Thermal Interface Materials. Polymers.

[79] Tutika R., Zhou S.H., Napolitano R.E., Bartlett M.D. (2018). Mechanical and Functional Tradeoffs in Multiphase Liquid Metal, Solid Particle Soft Composites. Adv. Funct. Mater..

[80] Hussain A.R.J., Alahyari A.A., Eastman S.A., Thibaud-Erkey C., Johnston S., Sobkowicz M.J. (2017). Review of Polymers for Heat Exchanger Applications: Factors Concerning Thermal Conductivity. Appl. Therm. Eng..

[81] Alam M.N., Kumar V., Jeong S.-U., Park S.-S. (2024). The Effect of Rubber–Metal Interactions on the Mechanical, Magneto–Mechanical, and Electrical Properties of Iron, Aluminum, and Hybrid Filler-Based Styrene–Butadiene Rubber Composites. Polymers.

[82] Tong X., Li N., Zeng M., Wang Q. (2019). Organic Phase Change Materials Confined in Carbon-Based Materials for Thermal Properties Enhancement: Recent Advancement and Challenges. Renew. Sustain. Energy Rev..

[83] Chen X., Cheng P., Tang Z., Xu X., Gao H., Wang G. (2021). Carbon-Based Composite Phase Change Materials for Thermal Energy Storage, Transfer, and Conversion. Adv. Sci..

[84] Shi X., Sun S., Zhao A., Zhang H., Zuo M., Song Y., Zheng Q. (2021). Influence of Carbon Black on the Payne Effect of Filled Natural Rubber Compounds. Compos. Sci. Technol..

[85] Song J., Tian K., Ma L., Li W., Yao S. (2019). The Effect of Carbon Black Morphology to the Thermal Conductivity of Natural Rubber Composites. Int. J. Heat Mass Transf..

[86] Bijina V., Jandas P.J., Joseph S., Gopu J., Abhitha K., John H. (2023). Recent Trends in Industrial and Academic Developments of Green Tyre Technology. Polym. Bull..

[87] Lu Y., Liu J., Hou G., Ma J., Wang W., Wei F., Zhang L. (2016). From Nano to Giant? Designing Carbon Nanotubes for Rubber Reinforcement and Their Applications for High Performance Tires. Compos. Sci. Technol..

[88] He Q., Zhou Y., Qu W., Zhang Y., Song L., Li Z. (2019). Wear Property Improvement by Short Carbon Fiber as Enhancer for Rubber Compound. Polym. Test..

[89] Chen Z., Tu Q., Shen X., Fang Z., Bi S., Yin Q., Zhang X. (2023). Enhancing the Thermal and Mechanical Properties of Carbon Fiber/Natural Rubber Composites by Co-Modification of Dopamine and Silane Coupling Agents. Polym. Test..

[90] Song J., Ma L., He Y., Yan H., Wu Z., Li W. (2015). Modified Graphite Filled Natural Rubber Composites with Good Thermal Conductivity. Chin. J. Chem. Eng..

[91] Tan X., Yuan Q., Qiu M., Yu J., Jiang N., Lin C.-T., Dai W. (2022). Rational Design of Graphene/Polymer Composites with Excellent Electromagnetic Interference Shielding Effectiveness and High Thermal Conductivity: A Mini Review. J. Mater. Sci. Technol..

[92] Sethulekshmi A.S., Saritha A., Joseph K. (2022). A Comprehensive Review on the Recent Advancements in Natural Rubber Nanocomposites. Int. J. Biol. Macromol..

[93] Xing W., Tang M., Wu J., Huang G., Li H., Lei Z., Fu X., Li H. (2014). Multifunctional Properties of Graphene/Rubber Nanocomposites Fabricated by a Modified Latex Compounding Method. Compos. Sci. Technol..

[94] Ouyang Y., Bai L., Tian H., Li X., Yuan F. (2022). Recent Progress of Thermal Conductive Ploymer Composites: Al2O3 Fillers, Properties and Applications. Compos. Part A Appl. Sci. Manuf..

[95] Song J., Peng Z., Zhang Y. (2020). Enhancement of Thermal Conductivity and Mechanical Properties of Silicone Rubber Composites by Using Acrylate Grafted Siloxane Copolymers. Chem. Eng. J..

[96] Wu Y., Ye K., Liu Z., Wang B., Yan C., Wang Z., Lin C.-T., Jiang N., Yu J. (2019). Cotton Candy-Templated Fabrication of Three-Dimensional Ceramic Pathway Within Polymer Composite for Enhanced Thermal Conductivity. ACS Appl. Mater. Interfaces.

[97] He S., Hu J., Zhang C., Wang J., Chen L., Bian X., Lin J., Du X. (2018). Performance Improvement in Nano-Alumina Filled Silicone Rubber Composites by Using Vinyl Tri-Methoxysilane. Polym. Test..

[98] Ouyang Y., Li X., Tian H., Bai L., Yuan F. (2021). A Novel Branched Al_2_O_3_/Silicon Rubber Composite with Improved Thermal Conductivity and Excellent Electrical Insulation Performance. Nanomaterials.

[99] Porrawatkul P., Nuengmatcha P., Kuyyogsuy A., Pimsen R., Rattanaburi P. (2023). Effect of Na and Al Doping on ZnO Nanoparticles for Potential Application in Sunscreens. J. Photochem. Photobiol. B.

[100] Zhang L., Zhang H., Chen C., Hu Z., Wang J. (2023). Preparation and Mechanism of High-Performance Ammonia Sensor Based on Tungsten Oxide and Zinc Oxide Composite at Room Temperature. Curr. Appl. Phys..

[101] Nandhini J., Karthikeyan E., Rajeshkumar S. (2024). Green Synthesis of Zinc Oxide Nanoparticles: Eco-Friendly Advancements for Biomedical Marvels. Resour. Chem. Mater..

[102] Abou Zeid S., Leprince-Wang Y. (2024). Advancements in ZnO-Based Photocatalysts for Water Treatment: A Comprehensive Review. Crystals.

[103] Mostoni S., Milana P., Di Credico B., D’Arienzo M., Scotti R. (2019). Zinc-Based Curing Activators: New Trends for Reducing Zinc Content in Rubber Vulcanization Process. Catalysts.

[104] Jeong M.W., Jeon S.W., Lee S.H., Kim Y. (2015). Effective Heat Dissipation and Geometric Optimization in an LED Module with Aluminum Nitride (AlN) Insulation Plate. Appl. Therm. Eng..

[105] Alsaad A.M., Al-Bataineh Q.M., Qattan I.A., Ahmad A.A., Ababneh A., Albataineh Z., Aljarrah I.A., Telfah A. (2020). Measurement and Ab Initio Investigation of Structural, Electronic, Optical, and Mechanical Properties of Sputtered Aluminum Nitride Thin Films. Front. Phys..

[106] Chiu H.T., Sukachonmakul T., Kuo M.T., Wang Y.H., Wattanakul K. (2014). Surface Modification of Aluminum Nitride by Polysilazane and Its Polymer-Derived Amorphous Silicon Oxycarbide Ceramic for the Enhancement of Thermal Conductivity in Silicone Rubber Composite. Appl. Surf. Sci..

[107] Liu X., Han Q., Yang D., Ni Y., Yu L., Wei Q., Zhang L. (2020). Thermally Conductive Elastomer Composites with Poly(Catechol-Polyamine)-Modified Boron Nitride. ACS Omega.

[108] Sarkarat M., Lanagan M., Ghosh D., Lottes A., Budd K., Rajagopalan R. (2020). Improved Thermal Conductivity and AC Dielectric Breakdown Strength of Silicone Rubber/BN Composites. Compos. Part C Open Access.

[109] Fang H., Bai S.-L., Wong C.P. (2016). “White Graphene”—Hexagonal Boron Nitride Based Polymeric Composites and Their Application in Thermal Management. Compos. Commun..

[110] An D., Chen H., He R., Chen J., Liu C., Sun Z., Yu H., Liu Y., Wong C., Feng W. (2024). MOF Decorated Boron Nitride/Natural Rubber Composites with Heterostructure for Thermal Management Application through Dual Passive Cooling Modes Base on the Improved Thermal Conductivity and Water Sorption-Desorption Process. Compos. Sci. Technol..

[111] Zhou X., Zong J., Lei J., Li Z. (2022). Enhancing Thermal Conductivity of Silicone Rubber via Constructing Hybrid Spherical Boron Nitride Thermal Network. J. Appl. Polym. Sci..

[112] Yang D., Kong X., Ni Y., Gao D., Yang B., Zhu Y., Zhang L. (2019). Novel Nitrile-Butadiene Rubber Composites with Enhanced Thermal Conductivity and High Dielectric Constant. Compos. Part A Appl. Sci. Manuf..

[113] Li A., Wang J., He W., Wei Z., Wang X., He Q. (2023). Enhancing Mechanical Property and Thermal Conductivity of Fluororubber by the Synergistic Effect of CNT and BN. Diam. Relat. Mater..

[114] Yang D., Ni Y., Kong X., Gao D., Wang Y., Hu T., Zhang L. (2019). Mussel-Inspired Modification of Boron Nitride for Natural Rubber Composites with High Thermal Conductivity and Low Dielectric Constant. Compos. Sci. Technol..

[115] Wang L., Shi Y., Sa R., Ning N., Wang W., Tian M., Zhang L. (2016). Surface Modification of Aramid Fibers by Catechol/Polyamine Codeposition Followed by Silane Grafting for Enhanced Interfacial Adhesion to Rubber Matrix. Ind. Eng. Chem. Res..

[116] Yang D., Wei Q., Yu L., Ni Y., Zhang L. (2021). Natural Rubber Composites with Enhanced Thermal Conductivity Fabricated via Modification of Boron Nitride by Covalent and Non-Covalent Interactions. Compos. Sci. Technol..

[117] Kuang Z., Chen Y., Lu Y., Liu L., Hu S., Wen S., Mao Y., Zhang L. (2015). Fabrication of Highly Oriented Hexagonal Boron Nitride Nanosheet/Elastomer Nanocomposites with High Thermal Conductivity. Small.

[118] Yang Y., Huang L., Dai Q., Cui L., Liu S., Qi Y., Dong W., He J., Bai C. (2019). Fabrication of β-Cyclodextrin-Crosslinked Epoxy Polybutadiene/Hydroxylated Boron Nitride Nanocomposites with Improved Mechanical and Thermal-Conducting Properties. J. Mater. Res. Technol..

[119] Harikrishnan R., Anirudh Mohan T.P., Rahulan N., Gopalan S. (2021). Effect of Silicon Nitride on Physical Properties of SBR. Mater. Today Proc..

[120] Heimann R.B. (2021). Silicon Nitride, a Close to Ideal Ceramic Material for Medical Application. Ceramics.

[121] Derradji M., Ramdani N., Zhang T., Wang J., Lin Z., Yang M., Xu X., Liu W. (2015). High Thermal and Thermomechanical Properties Obtained by Reinforcing a Bisphenol-A Based Phthalonitrile Resin with Silicon Nitride Nanoparticles. Mater. Lett..

[122] Ramdani N., Derradji M., Feng T., Tong Z., Wang J., Mokhnache E.-O., Liu W. (2015). Preparation and Characterization of Thermally-Conductive Silane-Treated Silicon Nitride Filled Polybenzoxazine Nanocomposites. Mater. Lett..

[123] Rasouli S., Zabihi A., Fasihi M., Kharat G.B.P. (2023). A Comprehensive Study on the Effect of Highly Thermally Conductive Fillers on Improving the Properties of SBR/BR-Filled Nano-Silicon Nitride. ACS Omega.

[124] Wang Y. (2022). Synthesis, Properties, and Multifarious Applications of SiC Nanoparticles: A Review. Ceram. Int..

[125] Yao Y., Zhu X., Zeng X., Sun R., Xu J.-B., Wong C.-P. (2018). Vertically Aligned and Interconnected SiC Nanowire Networks Leading to Significantly Enhanced Thermal Conductivity of Polymer Composites. ACS Appl. Mater. Interfaces.

[126] Bozorg Panah Kharat G., Zabihi A., Rasouli S., Fasihi M., Taki K. (2023). Accelerating the Kinetics of Curing Reaction of SBR/BR Blend by Silicon Carbide via Modification of Thermal Diffusivity. Mater. Today Commun..

[127] Weng G., Huang G., Qu L., Zhang P., Nie Y., Wu J. (2010). Natural Rubber with Low Heat Generation Achieved by the Inclusion of Boron Carbide. J. Appl. Polym. Sci..

[128] Hegde M., Chandrashekar A., Gopi J.A., Prabhu T.N. (2025). A Nanobridge Strategy to Fabricate Multifunctional Silicone Rubber Nanocomposites with Synergy Interplay in Enhancing Thermal Conductivity via BNNS-GO-PDA@MWCNT Ternary Fillers. J. Ind. Eng. Chem..

[129] Song S., Zhang Y. (2017). Carbon Nanotube/Reduced Graphene Oxide Hybrid for Simultaneously Enhancing the Thermal Conductivity and Mechanical Properties of Styrene-Butadiene Rubber. Carbon.

[130] Yu L., Yang D., Wei Q., Zhang L. (2021). Constructing of Strawberry-like Core-Shell Structured Al_2_O_3_ Nanoparticles for Improving Thermal Conductivity of Nitrile Butadiene Rubber Composites. Compos. Sci. Technol..

[131] Xie X., Yang D. (2023). Construction of Thermal Conduction Networks and Decrease of Interfacial Thermal Resistance for Improving Thermal Conductivity of Epoxy Natural Rubber Composites. Ceram. Int..

[132] Zhuang C., Tao R., Liu X., Zhang L., Cui Y., Liu Y., Zhang Z. (2021). Enhanced Thermal Conductivity and Mechanical Properties of Natural Rubber-Based Composites Co-Incorporated with Surface Treated Alumina and Reduced Graphene Oxide. Diam. Relat. Mater..

[133] Song J., Zhang Y. (2020). Vertically Aligned Silicon Carbide Nanowires/Reduced Graphene Oxide Networks for Enhancing the Thermal Conductivity of Silicone Rubber Composites. Compos. Part A Appl. Sci. Manuf..

[134] Yang D., Ni Y., Liang Y., Li B., Ma H., Zhang L. (2019). Improved Thermal Conductivity and Electromechanical Properties of Natural Rubber by Constructing Al_2_O_3_-PDA-Ag Hybrid Nanoparticles. Compos. Sci. Technol..

[135] Li H., Chen W., Xu J., Li J., Gan L., Chu X., Yao Y., He Y., Li B., Kang F. (2019). Enhanced Thermal Conductivity by Combined Fillers in Polymer Composites. Thermochim. Acta.

[136] Wang F., Zhou W., He Y., Lv Y., Wang Y., Wang Z. (2024). Synergetic Improvement of Dielectric Properties and Thermal Conductivity in Zn@ZnO/Carbon Fiber Reinforced Silicone Rubber Dielectric Elastomers. Compos. Part A Appl. Sci. Manuf..

[137] Li J., Zhao X., Zhang Z., Xian Y., Lin Y., Ji X., Lu Y., Zhang L. (2020). Construction of Interconnected Al_2_O_3_ Doped rGO Network in Natural Rubber Nanocomposites to Achieve Significant Thermal Conductivity and Mechanical Strength Enhancement. Compos. Sci. Technol..

[138] An D., Cheng S., Zhang Z., Jiang C., Fang H., Li J., Liu Y., Wong C.-P. (2019). A Polymer-Based Thermal Management Material with Enhanced Thermal Conductivity by Introducing Three-Dimensional Networks and Covalent Bond Connections. Carbon.

[139] Wei Q., Yang D., Yu L., Ni Y., Zhang L. (2020). Fabrication of Carboxyl Nitrile Butadiene Rubber Composites with High Dielectric Constant and Thermal Conductivity Using Al_2_O_3_@PCPA@GO Hybrids. Compos. Sci. Technol..

[140] Tang Y., Ma L., He Y., Chen H., Jiang Y., Xu J. (2019). Preparation and Performance Evaluation of Natural Rubber Composites with Aluminum Nitride and Aligned Carbon Nanotubes. Polym. Sci. Ser. A.

[141] Zhang K., Qiu J., Sakai E., Zhang G., Wu H., Guo S., Zhang L., Yamaguchi H., Chonan Y. (2024). Preparation of Continuous Carbon Fiber-filled Silicone Rubber with High Thermal Conductivity through Wrapping. Polym. Compos..

[142] Ji X., Wang Z., Wang J., Ye N., Zhang H., Lu Z., Li J., Lu Y. (2024). Mimicking Swallow Nest Structure to Construct 3D rGO/BN Skeleton for Enhancing the Thermal Conductivity of the Silicone Rubber Composites. Compos. Sci. Technol..

[143] Duan X., Tao R., Chen Y., Zhang Z., Zhao G., Liu Y., Cheng S. (2022). Improved Mechanical, Thermal Conductivity and Low Heat Build-up Properties of Natural Rubber Composites with Nano-Sulfur Modified Graphene Oxide/Silicon Carbide. Ceram. Int..

[144] Wei L., Fu X., Luo M., Xie Z., Huang C., Zhou J., Zhu Y., Huang G., Wu J. (2018). Synergistic Effect of CB and GO/CNT Hybrid Fillers on the Mechanical Properties and Fatigue Behavior of NR Composites. RSC Adv..

[145] Kodal M., Yazıcı Çakır N., Yıldırım R., Karakaya N., Özkoç G. (2023). Improved Heat Dissipation of NR/SBR-Based Tire Tread Compounds via Hybrid Fillers of Multi-Walled Carbon Nanotube and Carbon Black. Polymers.

[146] Wang T., Li M., Wu Z., Teng J., Xu J., Ying H., Xiong W., Zhu C. (2025). Preparation of a Precursor Complex Containing Lignin/Silica Hybrids and Styrene-Butadiene Rubber via a One-Pot Method to Fabricate High-Performance Rubber Materials. Int. J. Biol. Macromol..

[147] Wang J., Li S., Yang L., Liu B., Xie S., Qi R., Zhan Y., Xia H. (2024). Graphene-Based Hybrid Fillers for Rubber Composites. Molecules.

[148] Mirizzi L., D’Arienzo M., Nisticò R., Fredi G., Diré S., Callone E., Dorigato A., Giannini L., Guerra S., Mostoni S. (2023). Al_2_O_3_ Decorated with Polyhedral Silsesquioxane Units: An Unconventional Filler System for Upgrading Thermal Conductivity and Mechanical Properties of Rubber Composites. Compos. Sci. Technol..

[149] Dadkhah M., Messori M. (2024). A Comprehensive Overview of Conventional and Bio-Based Fillers for Rubber Formulations Sustainability. Mater. Today Sustain..

[150] Roy K., Debnath S.C., Potiyaraj P. (2020). A Review on Recent Trends and Future Prospects of Lignin Based Green Rubber Composites. J. Polym. Environ..

[151] Khan A., Kian L.K., Jawaid M., Khan A.A.P., Marwani H.M., Alotaibi M.M., Asiri A.M. (2022). Preparation and Characterization of Lignin/Nano Graphene Oxide/Styrene Butadiene Rubber Composite for Automobile Tyre Application. Int. J. Biol. Macromol..

[152] Mirizzi L., Carnevale M., D’Arienzo M., Milanese C., Di Credico B., Mostoni S., Scotti R. (2021). Tailoring the Thermal Conductivity of Rubber Nanocomposites by Inorganic Systems: Opportunities and Challenges for Their Application in Tires Formulation. Molecules.

[153] Tang T., Johnson D., Smith R.E., Felicelli S.D. (2014). Numerical Evaluation of the Temperature Field of Steady-State Rolling Tires. Appl. Math. Model..

[154] Behnke R., Kaliske M. (2015). Thermo-Mechanically Coupled Investigation of Steady State Rolling Tires by Numerical Simulation and Experiment. Int. J. Non-Linear Mech..

[155] Zeng Q.H., Yu A.B., Lu G.Q. (2008). Multiscale Modeling and Simulation of Polymer Nanocomposites. Prog. Polym. Sci..

[156] Zhao X., Fu B., Zhang W., Li H., Lu Y., Gao Y., Zhang L. (2020). Increasing the Thermal Conductivity of Styrene Butadiene Rubber: Insights from Molecular Dynamics Simulation. RSC Adv..

[157] Yang H., Cai F., Luo Y., Ye X., Zhang C., Wu S. (2020). The Interphase and Thermal Conductivity of Graphene Oxide/Butadiene-Styrene-Vinyl Pyridine Rubber Composites: A Combined Molecular Simulation and Experimental Study. Compos. Sci. Technol..

[158] Wang Z., Li Z., Lin X., Zheng L., Li Z., Lu S. (2025). Preparation and Molecular Simulation of Hyper-Dispersant Modified BN Filled Natural Rubber Thermally Conductive Composite. Polymer.

[159] Yang W., Lin Y., Zhu Y., Zhen C., Tao W., Luo Y., Wang X. (2025). Synergistic Effect of Dual-Modification Strategy on Thermal Conductivity and Thermal Stability of h-BN/Silicone Rubber Composites: Experiments and Simulations. Int. Commun. Heat Mass Transf..

[160] Fu Q., Yang Z., Jia H., Wen Y., Luo Y., Ding L. (2023). Integration of Experimental Methods and Molecular Dynamics Simulations for a Comprehensive Understanding of Enhancement Mechanisms in Graphene Oxide (GO)/Rubber Composites. J. Polym. Res..

[161] Liang X., Zhang X., Zhang L., Liu L., Du J., Zhu X., Ng K.M. (2019). Computer-Aided Polymer Design: Integrating Group Contribution and Molecular Dynamics. Ind. Eng. Chem. Res..

